# Validation of Risk Prediction Models to Detect Asymptomatic Carotid Stenosis

**DOI:** 10.1161/JAHA.119.014766

**Published:** 2020-04-20

**Authors:** Michiel H. F. Poorthuis, Alison Halliday, M. Sofia Massa, Paul Sherliker, Rachel Clack, Dylan R. Morris, Robert Clarke, Gert J. de Borst, Richard Bulbulia, Sarah Lewington

**Affiliations:** ^1^ Clinical Trial Service Unit and Epidemiological Studies Unit Nuffield Department of Population Health University of Oxford, United Kingdom; ^2^ MRC Population Health Research Unit Nuffield Department of Population Health University of Oxford, United Kingdom; ^3^ Department of Vascular Surgery University Medical Center Utrecht Utrecht The Netherlands; ^4^ Nuffield Department of Surgical Sciences John Radcliffe Hospital University of Oxford United Kingdom

**Keywords:** atherosclerosis, carotid artery stenosis, external validation, ischemic stroke, prevention, risk prediction model, targeted screening, Ischemic Stroke, Risk Factors, Primary Prevention, Stenosis, Atherosclerosis

## Abstract

**Background:**

Significant asymptomatic carotid stenosis (ACS) is associated with higher risk of strokes. While the prevalence of moderate and severe ACS is low in the general population, prediction models may allow identification of individuals at increased risk, thereby enabling targeted screening. We identified established prediction models for ACS and externally validated them in a large screening population.

**Methods and Results:**

Prediction models for prevalent cases with ≥50% ACS were identified in a systematic review (975 studies reviewed and 6 prediction models identified [3 for moderate and 3 for severe ACS]) and then validated using data from 596 469 individuals who attended commercial vascular screening clinics in the United States and United Kingdom. We assessed discrimination and calibration. In the validation cohort, 11 178 (1.87%) participants had ≥50% ACS and 2033 (0.34%) had ≥70% ACS. The best model included age, sex, smoking, hypertension, hypercholesterolemia, diabetes mellitus, vascular and cerebrovascular disease, measured blood pressure, and blood lipids. The area under the receiver operating characteristic curve for this model was 0.75 (95% CI, 0.74–0.75) for ≥50% ACS and 0.78 (95% CI, 0.77–0.79) for ≥70% ACS. The prevalence of ≥50% ACS in the highest decile of risk was 6.51%, and 1.42% for ≥70% ACS. Targeted screening of the 10% highest risk identified 35% of cases with ≥50% ACS and 42% of cases with ≥70% ACS.

**Conclusions:**

Individuals at high risk of significant ACS can be selected reliably using a prediction model. The best‐performing prediction models identified over one third of all cases by targeted screening of individuals in the highest decile of risk only.

**Clinical Perspective**

**What Is New?**

- Established risk prediction models to detect cases at high risk of asymptomatic carotid stenosis were validated in a contemporary screening population in the United States and United Kingdom.

**What Are the Clinical Implications?**

- Risk prediction models can be used for targeted screening for asymptomatic carotid stenosis, and cardiovascular risk management can be initiated or intensified to prevent complications of asymptomatic carotid stenosis.

**Nonstandard Abbreviations and Acronyms**

**ACS** asymptomatic carotid stenosis

**DBP** diastolic blood pressure

**HDL‐C** high‐density lipoprotein cholesterol

**LDL‐C** low‐density lipoprotein cholesterol

**SBP** systolic blood pressure

**TC** total cholesterol

**TIA** transient ischemic attack

## Introduction

Transient ischemic attack (TIA) or ischemic stroke is the first presentation of cardiovascular disease in about 25% of the cases,^1^, ^2^ and 15% to 20% of ischemic stroke cases are associated with extracranial carotid artery stenosis.^3^, ^4^, ^5^ Carotid stenosis is also a predictor for coronary events and vascular death.^6^ The prevalence of moderate (≥50%) and severe (≥70%) asymptomatic carotid stenosis (ACS) in the general population has been estimated to be 2.0% and 0.5%, respectively.^7^

Because of this low overall prevalence, population‐level screening for ACS with duplex ultrasound is not recommended in current guidelines.^8^, ^9^, ^10^, ^11^ However, targeted screening of high‐risk individuals might be worthwhile,^11^ and risk stratification tools or prediction models have been developed to provide individualized risk estimation for ACS. Before recommending targeted screening, risk prediction tools should be assessed for discrimination, calibration, and likely ability to detect false‐positive and false‐negative cases in an independent external population. We conducted a systematic review of published studies of prediction models for ACS and then externally validated these models in a large contemporary population of screenees in the United States and United Kingdom.

## Methods

Systematic review according to a predefined protocol to identify established risk prediction models. This protocol has been registered in an international registry for systematic reviews (PROSPERO [International Prospective Register of Systematic Reviews]): CRD42019108136. The study adhered to the Preferred Reporting Items for Systematic reviews and Meta‐Analyses (PRISMA) recommendations (Table S1) and the CHecklist for critical Appraisal and data extraction for systematic Reviews of prediction Modelling Studies (CHARMS).^12^, ^13^

### Data Sharing

Data from large population‐based studies conducted by the Nuffield Department of Population Health can be shared with bona fide researchers on application to the principal investigators of this study. Details of the departmental data access policy can be found at https://www.ndph.ox.ac.uk/data-access.

### Search Strategy and Eligibility Criteria

We used comprehensive electronic strategies and incorporated a validated research search filter to search Medline (via PubMed interface) and EMBASE (via OVID EMBASE interface) on March 1, 2019, for studies reporting on development and validation of prediction models for risk of significant ACS in general or screened populations (Data S1).^14^ We included studies that (1) addressed development and/or validation of diagnostic prediction models to detect ACS of 50% or greater, (2) assessed prediction models in both general and high‐risk populations but not in diseased populations at higher risk of ACS, (3) involved a cross‐sectional study design, and (4) were published in peer‐reviewed journals without any language restrictions.

### Screening Process and Data Extraction

Two authors (M.H.F.P. and M.S.M.) independently screened all titles and abstracts of the retrieved references and subsequently independently reviewed full‐text copies for final inclusion in this study. We performed backward citation searching using the bibliographies of included studies.

Two authors (M.H.F.P. and M.S.M.) independently extracted the following data from the included studies reporting the development of a prediction model, based on the CHARMS checklist: source of data, setting study, geographic area (country and continent), study years, sample size, modeling method (eg, logistic model), number of participants with missing data, handling of missing data, investigation of satisfaction of modeling assumptions, selection methods for predictor selection, shrinkage of predictor weights, number of outcome events, number of participants, degree of stenosis, number and type of predictors (diagnostic variables) used in the final model, number of outcome events per variable, presentation of model, and model performance (calibration and validation). In studies that reported internal validation of prediction models, we extracted the following additional data: method of internal validation (eg, cross‐validation, bootstrap), and whether the model was adjusted or updated after internal validation. In studies reporting external validation of a prediction model, we extracted the following additional data: type of external validation (eg, geographical and/or temporal distinct population), whether authors of the external validation also developed the original model, and performance of the model before or after model recalibration.

### Critical Appraisal

Prediction modeling studies were assessed for risk of bias and applicability using the Prediction model Risk Of Bias Assessment Tool (PROBAST).^15^ The assessment of risk of bias involved 4 domains: participants, predictors, outcome, and analysis. Risk of bias was judged as low, high, or uncertain for each domain. The assessment of applicability involved 3 domains: participants, predictors, and outcome. Applicability was judged as low, high, or uncertain for each domain. Each distinct model included in the article was evaluated separately.^16^

### External Validation Cohort

A cohort of 0.6 million self‐referred and self‐funded individuals who attended commercial vascular screening clinics between 2008 and 2013 in the United States and the United Kingdom was used for external validation. All individuals completed a standardized questionnaire including questions about their age; sex; height and weight; history of vascular disease (peripheral arterial disease, TIA, stroke, coronary artery disease, and congestive heart failure); history of hypertension; history of diabetes mellitus; smoking history; and use of antiplatelet, antihypertensive, and lipid‐lowering medication. Standard blood pressure cuffs and sphygmomanometers were used, with systolic pressure measured using a Doppler probe, and peripheral arterial disease was assessed with ankle‐brachial pressure index assessment.

Most participants underwent carotid duplex screening, conducted by trained staff using dedicated vascular ultrasound instruments (GE LOGIQ e). The highest peak systolic velocity and end‐diastolic velocity of both the common carotid arteries and the internal carotid arteries were measured.

A blood sample was collected from a subset of participants for selected plasma biochemical measurements using point‐of‐care testing methods (Alere Cholestech LDX System, Alere Inc, Waltham, MA). Plasma levels of total cholesterol, high‐density lipoprotein‐cholesterol, and triglycerides were measured by enzymatic methods. Low‐density lipoprotein‐cholesterol was estimated using the Friedewald formula (low‐density lipoprotein=total cholesterol−high‐density lipoprotein−triglycerides / 5).

### Predicted Outcomes

We externally validated the prediction models for both moderate or severe ACS:

1. Moderate or severe ACS; estimated stenosis of ≥50% (on the basis of peak systolic velocity ≥125 cm/s at either side or 0 cm/s for occluded arteries); and
2. Severe ACS, estimated stenosis of ≥70% (on the basis of peak systolic velocity ≥230 cm/s at either side or 0 cm/s for occluded arteries).

### Statistical Analysis (External Validation)

Selected characteristics of the external validation cohort were summarized using standard methods. We used the same external validation population for all external validation analyses to enable comparisons between different prediction models. Participants who provided a blood sample and had a duplex ultrasound performed were included in analyses. For most predictors, the percentage of participants with missing data was <12%, except for measured diastolic blood pressure (31.8%) (Table S2). Missing data were imputed using chained equations and we created 20 imputed data sets with 200 iterations.^17^ Total cholesterol/high‐density lipoprotein cholesterol ratio was calculated before imputation.^18^ Postimputation rounding was applied for limited‐range variables (systolic blood pressure, diastolic blood pressure, total cholesterol/high‐density lipoprotein cholesterol ratio, high‐density lipoprotein‐cholesterol, low‐density lipoprotein‐cholesterol, and height), if needed.^19^

The regression formula reported for each model was applied to the external validation cohort to calculate the probability of ≥50% and ≥70% ACS per participant. These individual probabilities were used for assessing the predictive performance. We contacted authors to provide the regression formula if it was not reported. If the authors did not report or could not provide the regression formula, we calculated a sum score (total points) for each participant by summing the scores assigned to each predictor in the original reports (referred to as a “score chart”). We used the sum score to assess the predictive performance.

We examined the performance of discrimination and calibration in the different prediction models. Discrimination is the ability of the prediction model to distinguish between participants with and without the disease outcomes, assessed using the area under the receiver operating characteristic (AUROC) curve. AUROC curve values were calculated per imputed data set and results were subsequently pooled using Rubin's rules.^20^, ^21^

Calibration is the agreement between predicted and observed risk and was assessed with calibration plots. For the models that provided the regression formula, we estimated the mean probability per participant across the 20 imputed data sets, and subsequently we split the predicted risks in deciles. We then calculated mean predicted and observed probability with corresponding 95% CIs per decile. In contrast, for the models that did not provide the regression formula, we used the predicted probability per sum score as reported in the original reports, and we calculated the observed probability with corresponding 95% CI in the validation cohort.

Differences between the prevalence of the predicted outcome in the development cohorts and the validation cohort are known to influence calibration. For this reason, we recalibrated the prediction models to the prevalence of the predicted outcome in the validation cohort by reestimating the intercept.^22^ We fitted a logistic model with a fixed calibration slope and the intercept as the only free parameter.^22^

STATA version 15.1 was used for all statistical analyses, and R version 3.5.1 was used for constructing the figures.

### Clinical Application

Clinical application of the prediction model with the best discrimination was assessed using 2 approaches. The first approach assessed targeted screening of the 10% and 20% cases at highest predicted risk of having significant ACS. For this, we calculated test characteristics for the highest decile and the highest 2 deciles of predicted risk. The second approach assessed targeted screening with a fixed level of sensitivity. For this, test characteristics were calculated for 2 levels of sensitivity (closest to sensitivity 80% and 90%).

### Sensitivity Analyses

We performed additional external validation of the prediction models: (1) in complete cases, (2) participants without a history of prior TIA or stroke using imputed data sets, and (3) participants without a history of prior cardiovascular disease (ie, stroke, TIA, myocardial infarction, and peripheral arterial disease) using imputed data sets.

### Ethical Approval

The University of Oxford Medical Sciences Inter‐Divisional Research Ethics Committee approved the study. All individuals provided written consent for the data collected at the screening visit to be used for research purposes.

### Role of the Funding Source

The study funders had no role in study design, data collection, analysis, or interpretation, drafting the report. The corresponding author had full access to all data in the study and had final responsibility for the decision to publish the report.

## Results

**Figure 1:**
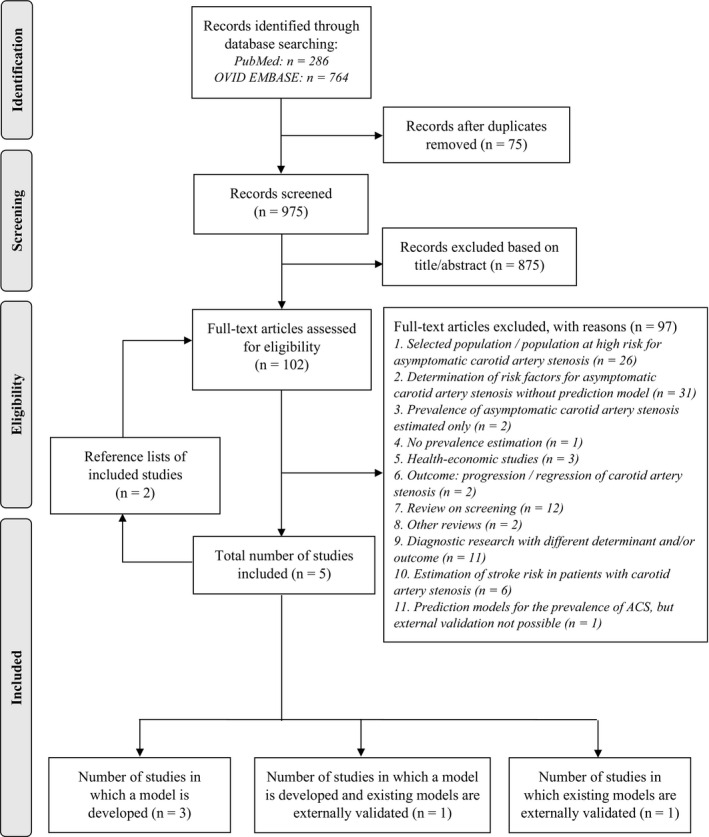
**Flowchart of literature review to identify the included studies.**

We screened 923 unique reports identified by literature searching, assessed the full texts of 102 reports for eligibility, and included 5 studies (Figure 1 and Table S3). Four studies involved model development studies, of which 1 performed additional external validation of an existing prediction model.^23^, ^24^, ^25^, ^26^ One study was an external validation study.^27^ Overall, 6 prediction models for the prevalence of significant ACS were developed.^23^, ^24^, ^25^, ^26^ Characteristics of model development are provided in Table 1 and Table S4.

**Table 1:** Selected Characteristics of Studies Assessing Different Risk Prediction Models for Significant ACS

	Predicted Outcomes	Data Sources	Calendar Year of Recruitment	No. of Cases/Participants in Derivation Cohort	Number of Included Predictors	Number of Events Per Predictor	First Author, Year of Publication
1.	70%‐100% ACS	Renqiu Stroke Screening Study, China	2012	18/3006 (0.6%)	7	2.6	Yan et al, 2018^26^ *Model 1*
2.	50%‐100% ACS			33/3006 (1.1%)	8	4.1	*Model 2*
3.	>70% ACS	Four observational studies: Sweden, Norway, Germany, four communities in the United States	Tromsø: 1994–1995; MDCS: 1991–1996; CAPS: NA; CHS: NA	127/23 706 (0.5%)	8	15.9	de Weerd et al, 2014^23^ *Model 1*
4.	>50% ACS			465/23 706 (2.0%)	8	58.1	*Model 2*
5.	>50% ACS	Screening, NY, USA	2001–2002	38/394 (9.6%)	4	9.5	Jacobowitz et al, 2003^24^
6.	≥60% ACS	Screening, NY, USA	1997	239/1331 (18%)	4	59.8	Qureshi et al, 2001^25^

ACS indicates asymptomatic carotid stenosis; CAPS, Carotid Atherosclerosis Progression Study; CHS, Cardiovascular Health Study; MDCS, Malmö Diet and Cancer Study; and NA, not available.

Three prediction models were developed to detect ACS ≥50%,^23^, ^24^, ^26^ 1 model was developed to detect ACS ≥60%,^25^ and 2 models were developed to detect ACS ≥70%.^23^, ^26^ The risk predictors included age, sex, smoking, hypertension, hypercholesterolemia, diabetes mellitus, myocardial infarction, stroke or TIA, height, measured blood pressure, and blood lipids. The number of predictors included in the prediction models varied from 4 to 8. Two models used clinical characteristics, and 4 models used blood measurements in addition to clinical characteristics. An overview of the predictors used in prediction models is provided in Table S5. The number of cases used to develop the prediction models varied from 394 to 23 706; the number of events varied from 18 to 465, and the number of cases per predictor varied from 2.6 to 59.8.

The overall risk of bias was low in 2 models and high in 4 models. Concerns with the applicability of the prediction models was deemed low in 3 models, unclear in 2 models, and high in 1 model. An overview of the risk of bias and the applicability per model is provided in Table S6.

### Predictive Performance

**Figure 2:**
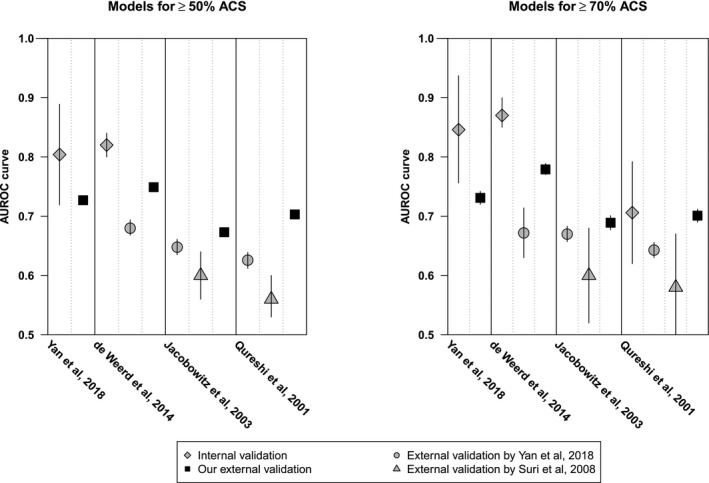
**Discriminative performance of risk prediction models.** The symbols represent the AUROC curves of the included prediction models and the vertical bars represent the 95% CIs. The values of the AUROC curves and 95% CIs are provided in Table S6. The models of Jacobowitz et al^24^ and Qureshi et al^25^ were originally developed for >50% ACS and ≥60% ACS, respectively. Suri et al, 2008 used ≥50% ACS and ≥75% ACS as outcomes for the external validation.^27^ The AUROC curves of 2 external validations for ≥50% ACS in the models developed for ≥70% ACS by de Weerd et al^23^ and Yan et al^26^ and 2 external validations for ≥70% ACS in the models developed for ≥50% ACS by the same authors are omitted in this figure. ACS indicates asymptomatic carotid stenosis; and AUROC, area under receiver operating characteristic.

Discriminative performance, as assessed by the AUROC curves varied from 0.81 to 0.88 in the derivation cohorts, and from 0.71 to 0.87 in the internal validation cohorts, respectively (Figure 2).^23^, ^24^, ^25^, ^26^, ^27^ Only 1 study provided calibration plots.^26^

In 2 studies, 10 external validation analyses were performed.^26^, ^27^ In Yan et al, 6 external validation analyses were performed using both ≥50% and ≥70% ACS as outcomes.^26^ The number of cases used for external validation in their study was 5010, of which 64 (1.3%) had ≥50% ACS, and 38 (0.8%) had ≥70% ACS. The AUROC curve ranged from 0.63 to 0.68. No (re)calibration was performed. A cohort from China used for external validation was geographically and temporally distinct from the derivation cohorts. In Suri et al, 4 external validation analyses were performed using ≥50% and ≥75% ACS as predicted outcomes.^27^ The number of cases used for external validation in their study was 5449, of which 227 (4.2%) had ≥50% ACS and 52 (1.0%) had ≥75% ACS. The AUROC curve ranged from 0.56 to 0.60. No (re)calibration was performed. The validation cohort was from the United States, as were the derivation cohorts of the validated models and the data of validation cohort were older than the derivation cohorts.

### External Validation

The validation cohort consisted of 596 469 participants, of whom 11 178 (1.87%) participants had ≥50% ACS and 2033 (0.34%) participants had ≥70% ACS. Baseline characteristics of the validation cohort are provided in Table 2.

**Table 2:** Selected Characteristics of Participants in the External Validation Cohort, by Severity of ACS

	Participants With <50% ACS (n=585 291)	Participants With 50% to 69% ACS (n=9145)	Participants With ≥70% ACS (n=2033)^b^	All Participants (n=596 469)
Age, y	62.0±10.0	68.7±8.9	68.3±8.8	62.2±10.1
Sex (male)	208 285 (35.6)	3442 (37.6)	1009 (49.6)	212 736 (35.7)
Current or former smoker	207 329 (40.0)	4865 (61.0)	1245 (69.2)	213 439 (40.4)
Never smoker	311 192 (60.0)	3112 (39.0)	555 (30.8)	314 859 (59.6)
Hypertension	202 768 (36.0)	5185 (58.9)	1166 (60.6)	209 119 (36.4)
Diabetes mellitus	44 986 (8.2)	1577 (18.3)	312 (16.4)	46 875 (8.4)
Coronary heart disease^a^	26 997 (5.1)	1262 (14.9)	344 (18.6)	28 603 (5.3)
Stroke/TIA	17 154 (3.3)	758 (9.0)	274 (15.0)	18 186 (3.4)
Peripheral arterial disease	16 370 (2.8)	1184 (13.4)	424 (21.8)	17 978 (3.1%)
Height, m	1.68±0.1	1.67±0.1	1.69±0.1	1.68±0.1
SBP, mm Hg	132±19.5	142±21.8	146±23.5	132±19.6
DBP, mm Hg	78±9.8	76±10.2	78±11.5	78±9.8
HDL‐C, mmol/L	1.4±0.5	1.3±0.5	1.3±0.4	1.4±0.5
LDL‐C, mmol/L	3.0±0.9	3.0±1.1	3.0±1.1	3.0±0.9
TC/HDL‐ratio	4.0±1.6	4.2±1.7	4.4±2.0	4.0±1.6

Values are mean±SD for continuous variables and n (%) for categorical variables. DBP indicates diastolic blood pressure; HDL‐C, high‐density lipoprotein cholesterol; LDL‐C, low‐density lipoprotein cholesterol; SBP, systolic blood pressure; TC, total cholesterol; and TIA, transient ischemic attack.

a In this group, 500 participants had a presumed occlusion.

b Coronary heart disease is defined as previous myocardial infarction or a coronary intervention (bypass, angioplasty, or stenting).

#### Discrimination for outcome ≥50% ACS

The model with the best discrimination showed an AUROC curve of 0.749 (95% CI, 0.744–0.753).^23^ The discriminative performance was fair in 3 other models with an AUROC curve of 0.727 (95% CI, 0.722–0.732), 0.704 (95% CI, 0.700–0.709) and 0.703 (95% CI, 0.699–0.708).^25^, ^26^ The discriminative performance was poor in 1 model with an AUROC curve of 0.673 (95% CI, 0.668–0.678).^24^

#### Discrimination for outcome ≥70% ACS

The model with the best discrimination showed an AUROC curve of 0.779 (95% CI, 0.770–0.789).^23^ The discriminative performance was fair in 3 other models with an AUROC curve of 0.759 (95% CI, 0.749–0.770), 0.731 (95% CI, 0.721–0.742) and 0.701 (95% CI, 0.690–0.712).^25^, ^26^ The discriminative performance was poor in 1 model with an AUROC curve of 0.689 (95% CI, 0.677–0.701)^24^ (Figure 2 and Table S7).

#### Calibration

**Figure 3:**
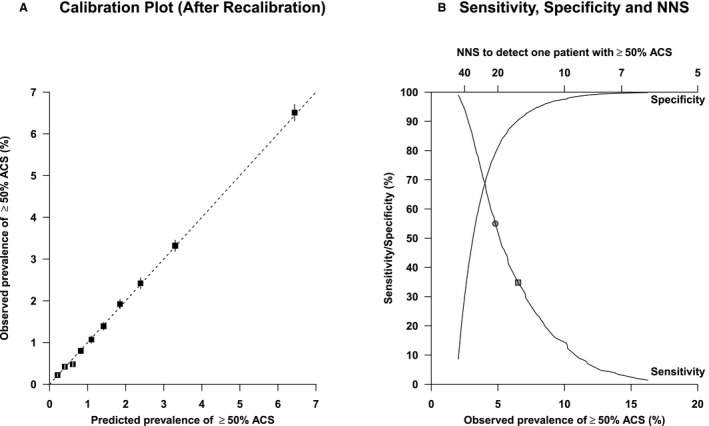
**Clinical application of the prediction model of de Weerd et al** 23 **for ≥50% ACS.** **A**, Calibration plot of external validation of the prediction model developed by de Weerd et al.^23^ It shows the predicted and observed prevalence of ≥50% ACS (after recalibration with adjusting the intercept). The boxes represent one decile of predicted risk, and the vertical lines represent the 95% CIs. **B**, Graph showing the sensitivity and specificity and corresponding observed prevalence and number needed to screen to detect 1 participant with ≥50% ACS using the prediction model developed by de Weerd et al.^23^ The square corresponds to targeted screening of participants in the highest decile of predicted risk. The prevalence in this decile is 6.5% with a number needed to screen of 15, and sensitivity is 34.8%. The circle corresponds to targeted screening of participants in the highest two deciles of predicted risk. The prevalence in these deciles is 4.8% with a number needed to screen of 21 and sensitivity of 55.0%. ACS indicates asymptomatic carotid stenosis; and NNS, number needed to scan.

In the model with the best discrimination, predicted probabilities (after recalibration with adjusting the intercept) showed good concordance between the predicted prevalence calculated with the prediction model and the observed prevalence in the external validation cohort. The predicted and observed prevalence of ≥50% ACS in the highest decile was 6.4% and 6.5%, respectively (Figure 3A).^23^ The predicted and observed prevalence of ≥70% ACS in the highest 2 deciles was 1.7% and 1.4%, respectively (Figure S1). Other calibration plots are provided as Figures S1 and S2 for the outcomes ≥70% ACS and ≥50% ACS, respectively.

### Application of the Prediction Model With the Best Discrimination

#### Application for outcome ≥50% ACS

First, we assessed targeted screening in the highest decile and highest 2 deciles of predicted risk. Prevalence of ≥50% ACS in the highest decile of predicted risk was 6.5% with a number needed to scan (NNS) of 15. Targeted screening of the highest decile identified 34.8% of cases with ≥50% ACS. Prevalence in the 2 highest deciles of predicted risk was 4.8% with an NNS of 21. Targeted screening of the 2 highest deciles identified 55.0% of cases with ≥50% ACS (Figure 3B and Table S8).

Second, we assessed targeted screening with fixed levels of sensitivity. For this, test characteristics were calculated for 2 levels of sensitivity (≈80% and 90%). Observed prevalences of ≥50% ACS were 2.78% and 3.38% for the sensitivity of 90.0% and 79.5%. The corresponding specificity was 40.0% and 56.6%, respectively (Table S8).

#### Application for outcome ≥70% ACS

Prevalence of ≥70% ACS in the highest decile of predicted risk was 1.4% with an NNS of 70. Targeted screening of the highest decile identified 41.7% of cases with ≥70% ACS. Prevalence in the 2 highest deciles of predicted risk was 0.98% with an NNS of 102. Targeted screening of the 2 highest deciles identified 62.1% of cases with ≥70% ACS (Figure S3 and Table S8).

Using fixed levels of sensitivity (≈80% and 90%), observed prevalences of ≥70% ACS were 0.8% and 0.5% for the sensitivity of 76.8% and 92.0%. The corresponding specificity was 65.1% and 40.0%, respectively (Table S8).

### Sensitivity Analysis

Validation in subsets with complete cases, cases without a history of TIA or stroke, showed comparable results. Validation in the subset of cases without a history of cardiovascular disease showed a lower AUROC (Figure S4 and Table S9).

## Discussion

The present study validated prediction models in an external population to identify a cohort of individuals at high risk of asymptomatic carotid stenosis (ACS). In the model with the best discrimination, the observed prevalence of ACS in the decile at highest risk was 6.5% (≥50% ACS) and 1.4% (≥70% ACS) with an NNS of 15 and 70, respectively. Targeted screening of individuals in the highest decile of risk reliably identified 35% of cases with ≥50% ACS and 42% of cases with ≥70% ACS.

Early identification of ACS cases allows the initiation or intensification of cardiovascular risk management using triple medical therapy (ie, antithrombotic, antihypertensive, and lipid‐lowering medication) to decrease the risk of cardiovascular disease. Carotid intervention might further decrease the risk of stroke in selected cases. Clinical and imaging features associated with an increased risk of stroke in patients with medically treated ACS, such as silent brain infarction, contralateral stroke, or TIA, plaque echolucency, intraplaque hemorrhage, microemboli, and reduced cerebrovascular reserve, have been identified.^10^, ^28^ Risk stratification tools, using a wide range of predictors, have been developed to estimate long‐term stroke and cardiovascular disease risk in cases with ACS, but these have not been validated with current medical treatment.^29^, ^30^ Reliable and validated risk stratification tools might help further refine the use of targeted screening for ACS by identifying cases at higher risk for stroke and cardiovascular disease.

We found that discrimination was less for participants without cardiovascular disease, but targeted screening could also include participants with a history of cerebrovascular or cardiovascular disease, since not all of these participants were taking adequate preventive treatments. Annual ipsilateral risk of stroke in ACS cases on medical therapy in previous randomized controlled trials varied between 1.4% and 2.4%.^31^, ^32^, ^33^ More recent studies have reported lower risks attributable to improving risk factor management.^29^ Annual risk of ipsilateral ischemic stroke and TIA in cases with >50% ACS and a history of TIA or minor stroke in another territory with consequent use of secondary prophylaxis was as low as 0.34% and 1.78%, respectively.^34^

The discrimination of the best model was fair and calibration good, despite differences between the original derivation and our validation cohort. Differences in duplex protocols, (eg, unilateral or bilateral screening), and differences in the methods of measurement of degree of stenosis between populations may have contributed to lower external performance in this large external validation cohort. Duplex screening does not assess intracranial stenosis, and extracranial calcified vessels can hamper reliable assessment. Different criteria for assessment of stenosis are available, but validity of duplex ultrasound performed by experienced sonographers is good,^35^ and peak systolic velocity, while it is a simple measurement, may be useful as a screening tool to identify cases for more intensive evaluation.

The present study had several strengths. We conducted an extensive literature search to identify existing models and previous external validation according to a prespecified protocol. We used a large cohort for external validation and all models were validated using the same participants, allowing us to directly compare their predictive performance. Missing data in the validation population were limited for most variables, and our findings were unaffected by missing values. Multiple imputation was used to handle missing data, which is preferred to complete‐case analysis. A direct match between predictors in the models and the external validation cohort was available for all predictors of externally validated models. Bilateral examination of the carotid arteries was performed and stenoses of either side were used as outcome. Our sensitivity analyses showed that exclusion of participants with previous stroke or TIA and exclusion of participants with previous cardiovascular disease did not influence the findings of the main analysis substantially.

The present study also had several limitations. First, even though the external validation data were prospectively collected, it was not primarily designed for research purposes. Second, participants were self‐referred and self‐funded, which may limit the generalizability to other (screened) populations. In addition, some predictors were not included in established risk prediction models, such as social status, possibly hampering reliable prediction in specific groups of patients. Third, data on medical history and height were assessed by self‐reporting and, hence, may be susceptible to recall bias. Fourth, data from duplex measurement of the internal carotid artery and common carotid artery were not recorded separately.

Risk prediction models with good calibration are needed to improve the efficiency of targeted screening programs by identifying those at greatest risk, but future research should determine the long‐term predictors of stroke and cardiovascular disease and determine the number of events that could be prevented by using more intensive medical treatment.

In conclusion, the present study showed that most prediction models had modest discrimination but could reliably identify a cohort of cases at high risk of ACS. The prevalence of ACS in the decile(s) at highest predicted risk of ACS was considerably higher than the overall prevalence, thereby substantially reducing the number of individuals needed to screen to detect ACS. Further research should determine the optimum thresholds required for a targeted screening by considering the number needed to screen, the diagnostic yield, the absolute reduction of stroke risk by prophylactic treatment, and cost‐effectiveness of different approaches.

## Sources of Funding

Prof Halliday is funded by the UK Health Research (NIHR) Oxford Biomedical Research Centre (BRC). Prof Lewington reports grants from UK Medical Research Council and from the CDC Foundation (with support from Amgen) outside the submitted work.

## Disclosures

None.

## Supporting information


**Data S1Tables S1–S9Figures S1–S4References 12, 14, 23–27, 36–131**
