# Supplementary material for: Validation of Risk Prediction Models to Detect Asymptomatic Carotid Stenosis
Source: J Am Heart Assoc. 2020 Apr 20;9(8):e014766. doi: 10.1161/JAHA.119.014766 (PMC7428515; doi:10.1161/JAHA.119.014766)
Supplement: Supplementary file 1 — Data S1Tables S1–S9Figures S1–S4References 12, 14, 23–27, 36–131 [file JAH3-9-e014766-s001.pdf]

Supplemental Material

Data S1.

Supplemental Methods. Search strategy Medline

(via PubMed interface)

1. "Carotid Stenosis"[Mesh]

2. "Carotid stenosis"[tiab] OR "Carotid artery stenosis"[tiab] OR "Carotid artery occlusion"[tiab] OR "Carotid artery stenoses"[tiab]

3. #1 OR #2

4. (Validat$ OR Predict$.ti. OR Rule$) OR (Predict$ AND (Outcome$ OR Risk$ OR Model$)) OR ((History OR Variable$ OR Criteria OR Scor$ OR Scoring$ OR Characteristic$ OR Finding$ OR Factor$) AND (Predict$ OR System$ OR Model$ OR Decision$ OR Identif$ OR Prognos$)) OR (Decision$ AND (Model$ OR Clinical$ OR Logistic Models/)) OR (Prognostic AND (History OR Variable$ OR Criteria OR Scor$ OR Characteristic$ OR Finding$ OR Factor$ OR Model$))[14]

5. "Mass Screening"[Mesh] OR Screen*[tiab]

6. Prevalence[Mesh] OR prevalenc* OR communit*[tiab]

7. "Population"[MeSH Terms] OR population*[tiab]

8. #5 OR #6 OR #7

9. #3 AND #4 AND #8

----

286 references identified on March 1, 2019

EMBASE (via OVID EMBASE interface) [*]

1. exp carotid artery stenosis/

2. (carotid artery or carotid artery atherosclerosis or carotid artery disease or carotid artery diseases).ti,ab,kw.

3. stenos*.ti,ab,tw.

4. 2 AND 3

5. 1 OR 4

6. predict.ti.

7. (validat* or rule*).ti,ab.

8. (predict* and (outcome* or risk* or model*)).ti,ab.

9. ((history or variable* or criteria or scor* or characteristic* or finding* or factor*) and (predict* or model* or decision* or identif* or prognos*)).ti,ab.

10. decision*.ti,ab. and statistical model/

*https://www.nice.org.uk/guidance/ng50/documents/search-strategies

11. (decision* and (model* or clinical*)).ti,ab.

12. (prognostic and (history or variable* or criteria or scor* or characteristic* or finding* or factor* or model*)).ti,ab.

13. (stratification or discrimination or discriminate or c statistic or "area under the curve" or auc or calibration or indices or algorithm or multivariable).ti,ab.

14. receiver operating characteristic/

15. 6 OR 7 OR 8 OR 9 OR 10 OR 11 OR 12 OR 13 OR 14

16. exp mass screening/

17. Screening.ab,ti,kw.

18. exp prevalence/

19. Prevalence.ab,ti,kw.

20. 16 OR 17 OR 18 OR 19

21. 5 AND 15 AND 20

22. letter.pt. or letter/

23. note.pt.

24. conference abstract.pt.

25. editorial.pt.

26. case report/ or case study/

27. (letter or comment*).ti.

28. 22 OR 23 OR 24 OR 25 OR 26 OR 27

29. animal/ not human/

30. nonhuman/

31. exp animal experiment/

32. exp experimental animal/

33. animal model/

34. exp rodent/

35. (rat or rats or mouse or mice).ti.

36. 29 or 30 or 31 or 32 or 33 or 34 OR 35

37. 28 OR 36

38. 21 NOT 37

----

764 references identified on March 1, 2019

Table S1. PRISMA checklist [12]

Section/topic	#	Checklist item	Reported
**TITLE**			
Title	1	Identification as a systematic review, meta-analysis, or both.	NA
**ABSTRACT**			
Structured summary	2	Structured abstract including background, objectives, data sources, study eligibility criteria, methodological assessment, synthesis method, results, conclusions and implications of key findings.	✓
**INTRODUCTION**			
Rationale	3	Describe the rationale for the review in the context of what is already known.	✓
Objectives	4	Provide an explicit statement of questions being addressed with reference to participants, interventions, outcomes (PICO design).	✓
**METHODS**			
Protocol and registration	5	Indicate if a review protocol exists, and where it can be accessed.	✓
Eligibility criteria	6	Specify study characteristics and report characteristics (such as years considered, language, publication status) used as criteria for eligibility.	✓
Information sources	7	Describe all information sources (such as databases with dates of coverage, contact with study authors, experts) in the search, and the date of last search.	✓
Search	8	Present full electronic search strategy, including limits used, such that it could be repeated.	✓
Study selection	9	State the process for selecting studies (i.e., screening, eligibility) and make sure that this is done by 2 authors.	✓
Data collection	10	Describe method of data extraction from reports (e.g., piloted forms, independently, in duplicate) and any processes for obtaining and confirming data from investigators.	✓
Data items	11	List and define all variables for which data were sought (e.g., PICOS, funding sources) and any assumptions and simplifications made.	✓
Risk of bias in individual studies	12	Describe methods used for assessing risk of bias of individual studies (including specification of whether this was done at the study or outcome level), and how this information is to be used in any data synthesis.	✓
Summary measures	13	State the principal summary measures (e.g., risk ratio, difference in means).	✓
Synthesis of results	14	Describe the methods of handling data and combining results of studies, if done, including measures of consistency (e.g., I^2) for each meta-analysis.	NA
Risk of bias across studies	15	Specify any assessment of risk of bias that may affect the cumulative evidence (e.g., publication bias, selective reporting within studies).	✓
Additional analyses	16	Describe methods of additional analyses (e.g., sensitivity or subgroup analyses, meta-regression), if done, indicating which were pre-specified.	✓
**RESULTS**			
Study selection	17	Give numbers of studies screened, assessed for eligibility, and included in the review, with reasons for exclusions at each stage, illustrated with a flow diagram.	✓
Study characteristics	18	For each study, present characteristics for which data were extracted (e.g., study size, PICOS, follow-up period) and provide the citations.	✓

Risk of bias within studies	19	Present data on risk of bias of each study and, if available, any outcome level assessment.	✓
Results of individual studies	20	For all outcomes considered (benefits or harms), present, for each study: (a) simple summary data for each intervention group (b) effect estimates and confidence intervals, ideally with a forest plot.	✓
Synthesis of results	21	Present results of each meta-analysis done, including confidence intervals and measures of consistency.	NA
Risk of bias across studies	22	Present results of any assessment of risk of bias across studies.	✓
Additional analysis	23	Give results of additional analyses, if done (e.g., sensitivity or subgroup analyses, meta-regression).	✓
**DISCUSSION**			
Summary of evidence	24	Summarize the main findings including the strength of evidence for each main outcome; consider their relevance to key groups (e.g., healthcare providers, users, and policy makers).	✓
Limitations	25	Discuss limitations at study and outcome level (e.g., risk of bias), and at review-level (e.g., incomplete retrieval of identified research, reporting bias).	✓
Conclusions	26	Provide a general interpretation of the results in the context of other evidence, and implications for future research.	✓
**FUNDING**			
Funding	27	Describe sources of funding for the systematic review and other support (e.g., supply of data); role of funders for the systematic review.	✓

Table S2. Missing data per variable

Variable	Percentage of participants with missing
Age	0
Sex	0
Current or former smoker	11.4
Never smoked	11.4
Hypertension	3.69
Diabetes mellitus	6.22
Coronary heart disease	8.91
Stroke/TIA	9.90
Peripheral arterial disease	1.70
Height	1.79
SBP	0.48
DBP	31.8
HDL-C	0.3
LDL-C	8.6
TC/HDL-ratio	0.3

DBP indicates diastolic blood pressure; HDL-C, high-density lipoprotein cholesterol; LDL-C, low-density lipoprotein cholesterol; SBP, systolic blood pressure; TC, total cholesterol; TIA, transient ischemic attack.

Table S3. Full-text evaluation

	First author, year of publication	Selected population/ diseased population at high risk for ACS	Determination of risk factors of ACS without prediction model	Prevalence of ACS estimated only	No prevalence estimation of ACS	Health-economic research on ACS	Outcome: progression/regression of carotid artery stenosis	Review on screening	Other reviews	Diagnostic research with different determinant and/or outcome	Estimation of stroke risk or mortality in patients with ACS	External validation not possible	**Included studies**
1.	Abd Allah et al, 2010[36]		*										
2.	Aboyans et al, 2009[37]	*											
3.	Admani et al, 1991[38]		*										
4.	Ahn et al, 1991[39]	*											
5.	Aizenberg, 2016[40]							*					
6.	Alexandrov, 2003[41]								*				
7.	Ansari et al, 2011[42]	*											
8.	Archbold et al, 2001[43]	*											
9.	Ascher et al, 2001[44]	*											
10.	Ballard et al, 2007[45]			*									
11.	Barvalia et al, 2014[46]	*											
12.	Belcaro et al, 2000[47]							*					
13.	Berens et al, 1992[48]	*											
14.	Berger et al, 2013[49]		*										
15.	Bosevski et al, 2007[50]	*											
16.	Bosevski et al, 2015[51]	*											
17.	Carnicelli et al, 2014[52]									*			
18.	Carnicelli et al, 2013[53]									*			
19.	Carsten et al, 1999[54]									*			
20.	Chiquete et al, 2014[55]	*											
21.	Chua et al, 2007[56]									*			
22.	Chou et al, 2018[57]		*										
23.	Colgan et al, 1988[58] †		*										
24.	Cull et al, 2011[59]				*								
25.	de Weerd et al, 2014[23]												*
26.	Derdeyn et al, 1996[60]					*							
27.	Derdeyn et al, 1995[61]									*			
28.	Di Carli et al, 2005[62]	*											
29.	Duval et al, 2006[63]	*											
30.	Ellis et al, 1992[64]	*											
31.	Elmore et al, 2003[65]		*										
32.	Engelhardt et al, 2005[66]									*			
33.	Eugene et al, 1999[67]								*				
34.	Fabris et al, 1994[68]		*										
35.	Felberg et al, 2002[69]									*			
36.	Fernandes et al, 2016[70]										*		
37.	Ghanaati et al, 2009[71]	*											
38.	Giral et al, 1999[72]									*			
39.	Gao et al, 2011[73]	*											
40.	Greco et al, 2013[74]											*	
41.	Hedblad et al, 1998[75]										*		
42.	Helfre et al, 2017[76]	*											
43.	Hogberg et al, 2014[77]		*										
44.	Hoshino et al, 2018[78]	*											
45.	Howard et al, 1996[79]									*			
46.	Hua et al, 2014[80]												
47.	Hughes et al, 2010[81]		*										

	First author, year of publication	Selected population/ diseased population at high risk for ACS	Determination of risk factors of ACS without prediction model	Prevalence of ACS estimated only	No prevalence estimation of ACS	Health-economic research on ACS	Outcome: progression/regression of carotid artery stenosis	Review on screening	Other reviews	Diagnostic research with different determinant and/or outcome	Estimation of stroke risk or mortality in patients with ACS	External validation not possible	**Included studies**
48.	Jacobowitz et al, 2003[24]												*
49.	Joakimsen et al, 2000[82]										*		
50.	Jonas et al, 2014[83]							*					
51.	Kakkos et al, 2014[84]						*						
52.	Karnon et al, 2007[85]							*					
53.	Kazemi-Bajestani et al, 2013[86]	*											
54.	Kazum et al, 2016[87]	*											
55.	Lacroix et al, 2006[88]	*											
56.	Lassila et al, 1997[89]		*										
57.	Lee et al, 1997[90]							*					
58.	LeFevre et al, 2014[91]							*					
59.	Li et al, 2013[92]	*											
60.	Liang et al, 2014[93]		*										
61.	Lim et al, 2011[94]							*					
62.	Lim et al, 2006[95]		*										
63.	Martin et al, 2004[96]	*											
64.	Mathiesen et al, 2001[97]		*										
65.	Meng et al, 2017[98]		*										
66.	Moneta et al, 1989[99]										*		
67.	Mostaza et al, 2009[100]	*											
68.	Niederkorn et al, 1991[101]		*										
69.	Obuchowski et al, 1997[102]							*					
70.	O'Leary et al, 1993[103]										*		
71.	O'Leary et al, 1992[104]		*										
72.	Paprottka et al, 2017[105]	*											
73.	Park et al, 2006[106]	*											
74.	Prati et al, 1992[107] †		*										
75.	Prati et al, 2006[108]		*										
76.	Qiu et al, 2016[109]		*										
77.	Qureshi et al, 2001[25]												*
78.	Rockman et al, 2013[110]		*										
79.	Rockman et al, 2004[111]		*										
80.	Rodriguez Saldana et al, 1998			*									
81.	Roh et al, 2011[112]		*										
82.	Ryglewicz et al, 1998[113]	*											
83.	Saleem et al, 2008[114]							*					
84.	Savji et al, 2013[115]		*										
85.	Shah et al, 2014[116]		*										
86.	Silaghi et al, 2013[117]		*										
87.	Smolen et al, 2007[118]										*		
88.	Solomon et al, 1997[119]							*					
89.	Stein et al, 2015[120]		*										
90.	Suri et al, 2008[27]												*
91.	Sutton-Tyrrel et al, 1993[121]		*										
92.	Touzé et al, 2008[122]							*					
93.	Walters et al, 1993[123]									*			
94.	Weisman et al, 2015[124]					*							
95.	Whitty et al, 1998[125]									*			
96.	Willeit et al, 1993[126]		*										
97.	Woo et al, 2017[127]		*										

	First author, year of publication	Selected population/ diseased population at high risk for ACS	Determination of risk factors of ACS without prediction model	Prevalence of ACS estimated only	No prevalence estimation of ACS	Health-economic research on ACS	Outcome: progression/regression of carotid artery stenosis	Review on screening	Other reviews	Diagnostic research with different determinant and/or outcome	Estimation of stroke risk or mortality in patients with ACS	External validation not possible	**Included studies**
98.	Wyman et al, 2006[128]							*					
99.	Yan et al, 2018[26]												*
100.	Yin et al, 1998[129]					*							
101.	Yu et al, 2009[130]		*										
102.	Zorach et al, 2016[131]						*						

† These articles were identified through cross-checking the reference lists of the studies included.
ACS indicates asymptomatic carotid artery stenosis.

Table S4. Characteristics of included model derivation and/or internal validation studies

	Predicted outcome	Data source	No. events / No. total patients	Modelling method	Handling of missing data	Selection methods for predictor selection	Correction for overoptimism	Number of predictive factors	Presentation of risk model	First author, year of publication
1.	≥70% ACS	Renqiu Stroke Screening Study, China	18 / 3006 (0.6%)	Logistic	No details provided	Backward	No	7	Regression coefficients and web calculator	Yan et al, 2018[26] *Model 1*
2.	≥50% ACS		33 / 3006 (1.1%)	Logistic	No details provided	Backward	No	8	Regression coefficients and web calculator	*Model 2*
3.	≥70% ACS	4 observational studies: Sweden, Norway, Germany, 4 communities in the US	127 / 23706 (0.5%)	Logistic	Imputation (single regression technique)	Based on the predictors for moderate stenosis	Yes	8	Original model, scoring chart	de Weerd et al, 2014[23] *Model 1*
4.	≥50% ACS		465 / 23706 (2.0%)	Logistic	Imputation (single regression technique)	Backward	Yes	8	Original model, scoring chart	*Model 2*
5.	>50% ACS	Screening, NY, US	38 / 394 (9.6%)	Logistic and X^2 analysis*	Not stated	Based on univariate analysis	No	4	Original model	Jacobowitz et al, 2003[24]
6.	≥60% ACS	Screening, NY, US	239 / 1331 (18%)	Logistic	Not stated	Based on univariate analysis	No	4	Original model	Qureshi et al, 2001[25]

ACS indicates asymptomatic carotid artery stenosis.

* Logistic regression and X^2 analysis were not used to weight the diagnostic variables in the prediction model.

Table S4. Characteristics of included model derivation and/or internal validation studies (continued)

	Discrimination		Calibration			First author, year of publication
	AUROC curve	Sensitivity / specificity	Calibration plot	Hosmer-Lemeshow test	Observed-expected ratio	
1.	0.806 (95% CI 0.724-0.889)	-	Yes	P > 0.05	High correlation between observed and predicted risk: r = 0.924, P < 0.001	Yan et al, 2018[26] *Model 1*†
2.	0.785 (95% CI 0.705-0.864)	-	Yes	P > 0.05	High correlation between observed and predicted risk: r = 0.955, P < 0.001	*Model 2*†
3.	0.87 (0.85-0.90)*	Yes	-	P = 0.071	-	de Weerd et al, 2014[23] *Model 1*‡
4.	0.82 (0.80-0.84)*	Yes	-	P = 0.585	-	*Model 2*‡
5.	-	-	-	-	-	Jacobowitz et al, 2003[24]
6.	0.706 (0.620-0.792)	-	-	-	-	Qureshi et al, 2001[25]

AUROC curve indicates area under receiver operating characteristic curve.
* This AUROC curve was calculated after bootstrapping techniques were applied. † Model 1 refers to the model that was developed with predicted outcome 70-100% ACS and model 2 refers to the model that was developed with predicted outcome 50-100% ACS. ‡ Model 1 refers to the model that was developed with predicted outcome >70% ACS and model 2 refers to the model that was developed with predicted outcome >50% ACS.

Table S5. Predictors (diagnostic variables) used in the prediction models

**Risk predictors**	**Yan et al, 2018[26] (Model: ≥50% ACS)**	**Yan et al, 2018[26] (Model: ≥70% ACS)**	**De Weerd et al, 2014[23] (Both models)**	**Jacobowitz et al, 2003[24]**	**Qureshi et al, 2001[25]**
Age*	*	*	*		*
Sex	*	*	*		
Current smoking			*	*	*
Hypertension				*	
Hypercholesterolemia				*	*
Diabetes mellitus			*		
History of stroke/TIA	*	*			
Coronary artery disease					*
Cardiac disease				*	
History of vascular disease†			*		
History of peripheral arterial disease	*	*			
Height (per cm increase)	*	*			
SBP‡	*		*		
DBP§	*		*		
HDL (per mmol/L increase)	*	*			
LDL (per mmol/L increase)		*			
TC/HDL ratio			*		

ACS indicates asymptomatic carotid stenosis; DBP, diastolic blood pressure; HDL-C, high-density lipoprotein cholesterol; LDL, low-density lipoprotein cholesterol; SBP, systolic blood pressure; TC, total cholesterol; TIA, transient ischemic attack.

* Age was defined as per year increase (in Yan et al, 2018), categorized in four groups (in de Weerd et al, 2014) and dichotomized in >65 years and ≤65 years (in Qureshi et al, 2001). † History of vascular disease is defined as a medical history of either coronary heart disease or stroke. ‡ SBP was defined as per mmHg increase (in Yan et al, 2018), categorized in three groups (in de Weerd et al, 2014). § DBP was defined as per mmHg increase (in Yan et al, 2018), categorized in three groups (in de Weerd et al, 2014).

Table S6. Risk of bias assessment using PROBAST

First author, year of publication	Risk of bias				Applicability			Overall	
	Participants	Predictors	Outcome	Analysis	Participants	Predictors	Outcome	Risk of bias	Applicability
1. Yan et al, 2018[26] *Model 1**	-	?	?	-	+	+	?	-	?
2. *Model 2**	-	?	?	-	+	+	?	-	?
3. de Weerd et al, 2014[23] *Model 1†*	+	+	+	+	+	+	+	+	+
4. *Model 2†*	+	+	+	+	+	+	+	+	+
5. Jacobowitz et al, 2003[24]	-	?	+	-	-	+	+	-	-
6. Qureshi et al, 2001[25]	+	+	+	-	+	+	+	-	+

PROBAST indicates Prediction model Risk Of Bias ASessement Tool.
+ indicates low risk of bias / low concern regarding applicability; - indicates high risk of bias / high concern regarding applicability; and ? indicates unclear risk of bias / unclear concern regarding applicability.
An overview of all steps per prediction model is available on request.
* Model 1 refers to the model that was developed with predicted outcome 70-100% ACS and model 2 refers to the model that was developed with predicted outcome 50-100% ACS. † Model 1 refers to the model that was developed with predicted outcome >70% ACS and model 2 refers to the model that was developed with predicted outcome >50% ACS.

Table S7. Discrimination of each prediction model in the original cohort and validation cohorts

	First author, year of publication	Predicted outcome in original model	Model development study: AUROC curve (95% CI) in derivation cohort	Model development study: AUROC curve (95% CI) in internal validation cohort	Previous external validations: Predicted outcome of external validation	Previous external validations: AUROC curve (95% CI)
1.	Yan et al, 2018[26] *Model 1**	70-100% ACS	0.785 (0.705-0.864)	0.846 (0.756-0.937)‡	-	-
2.	*Model 2**	50-100% ACS	0.806 (0.724-0.889)	0.804 (0.719-0.889)‡	-	-
3.	de Weerd et al, 2014[23] *Model 1†*	>70% ACS	-	0.87 (0.85-0.90)§	70-100% ACS	0.672 (0.630-0.657)[26]
4.	*Model 2†*	>50% ACS	-	0.82 (0.80-0.84)§	50-100% ACS	0.680 (0.668-0.694)[26]
5.	Jacobowitz et al, 2003[24]	>50% ACS	-	-	70-100% ACS	0.670 (0.657-0.683)[26]
					50-100% ACS	0.648 (0.635-0.661)[26]
					75-100% ACS	0.60 (0.52-0.68)[27]
					50-100% ACS	0.60 (0.56-0.64)[27]
6.	Qureshi et al, 2001[25]	≥60% ACS	-	0.706 (0.620-0.792)‖	70-100% ACS	0.643 (0.630-0.656)[26]
					50-100% ACS	0.626 (0.612-0.639)[26]
					75-100% ACS	0.58 (0.50-0.67)[27]
					50-100% ACS	0.56 (0.53-0.60)[27]

ACS indicates asymptomatic carotid artery stenosis; AUROC curve, area under receiver operating characteristic curve; CI, confidence interval.

* Model 1 refers to the model that was developed with predicted outcome 70-100% ACS and model 2 refers to the model that was developed with predicted outcome 50-100% ACS. † Model 1 refers to the model that was developed with predicted outcome >70% ACS and model 2 refers to the model that was developed with predicted outcome >50% ACS. ‡ Model was internally validated using split sample with random division of participants: 60% was assigned to the derivation cohort and 40% was assigned to the validation cohort. § Model was internally validated with bootstrapping techniques to correct for overoptimism. ‖ Model was internally validated using split sample with random division of participants after excluding patients with history of transient ischemic attack, stroke, or carotid artery surgery: 66% was used for the derivation cohort and 33% was used for the validation cohort.

Table S7. Discrimination of each prediction model in the original cohort and validation cohorts (continued)

	First author, year of publication	Predicted outcome in original model	Our external validation			
			Predicted outcome of our external validation	AUROC curve (95% CI)	Predicted outcome of our external validation	AUROC curve (95% CI)
1.	Yan et al, 2018[26] *Model 1**	70-100% ACS	≥50% ACS	0.704 (0.700-0.709)	≥70% ACS	0.731 (0.720-0.742)
2.	*Model 2**	50-100% ACS	≥50% ACS	0.727 (0.722-0.732)	≥70% ACS	0.759 (0.749-0.770)
3.	de Weerd et al, 2014[23] *Model 1†*	>70% ACS	≥50% ACS	0.749 (0.744-0.753)	≥70% ACS	0.779 (0.770-0.789)
4.	*Model 2†*	>50% ACS	≥50% ACS	0.749 (0.744-0.753)	≥70% ACS	0.779 (0.770-0.789)
5.	Jacobowitz et al, 2003[24]	>50% ACS	≥50% ACS	0.673 (0.668-0.678)	≥70% ACS	0.689 (0.677-0.701)
6.	Qureshi et al, 2001[25]	≥60% ACS	≥50% ACS	0.703 (0.699-0.708)	≥70% ACS	0.701 (0.690-0.712)

ACS indicates asymptomatic carotid artery stenosis; AUROC curve, area under receiver operating characteristic curve; CI, confidence interval.
* Model 1 refers to the model that was developed with predicted outcome 70-100% ACS and model 2 refers to the model that was developed with predicted outcome 50-100% ACS. † Model 1 refers to the model that was developed with predicted outcome >70% ACS and model 2 refers to the model that was developed with predicted outcome >50% ACS.

Table S8. Clinical application of the prediction model with the best discrimination ***Outcome ≥50% ACS***

**Sensitivity**	**Specificity**	**PPV**	**NPV**	**True positive**	**False negative**	**False positive**	**True negative**	**Observed prevalence**	**NNS**
Highest decile of predicted risk of ≥50% ACS									
**34.8%**	90.5%	6.51%	98.6%	3,885	7,293	55,762	529,529	6.51%	15
Highest two deciles of predicted risk of ≥50% ACS									
**55.0%**	79.2%	4.81%	98.9%	6,149	5,029	121,676	463,615	4.81%	21
Two different levels of sensitivity for the outcome ≥50% ACS									
**79.5%**	56.6%	3.38%	99.3%	8,882	2,296	254,033	331,258	3.38%	30
**90.0%**	40.0%	2.78%	99.5%	10,060	1,118	351,171	234,120	2.78%	36

ACS indicates asymptomatic carotid artery stenosis; NNS, number needed to screen; NPV, negative predictive value; PPV, positive predictive value.

Table S8. Clinical application of the prediction model with the best discrimination (continued)

Outcome ≥70% ACS

**Sensitivity**	**Specificity**	**PPV**	**NPV**	**True positive**	**False negative**	**False positive**	**True negative**	**Observed prevalenc e**	**NNS**
Highest decile of predicted risk of ≥70% ACS									
**41.7%**	90.1%	1.42%	99.8%	848	1,185	58,799	535,637	1.42%	70
Highest two deciles of predicted risk of ≥70% ACS									
**62.1%**	78.5%	0.98%	99.8%	1,263	770	127,566	466,870	0.98%	102
Two different levels of sensitivity for the outcome ≥70% ACS									
**76.8%**	65.1%	0.75%	99.9%	1,561	472	207,361	387,075	0.75%	133
**92.0%**	40.0%	0.52%	99.9%	1,870	163	356,506	237,930	0.52%	192

ACS indicates asymptomatic carotid artery stenosis; NNS, number needed to screen; NPV, negative predictive value; PPV, positive predictive value.

Table S9. Sensitivity analyses

	Prediction model	AUROC (95% CI) for ≥50% ACS			AUROC (95% CI) for ≥70% ACS		
		Complete-case analysis	Without patients with previous TIA or stroke	Without patients with previous CVD	Complete-case analysis	Without patients with previous TIA or stroke	Without patients with previous CVD
1	Yan et al, 2018[26] *Model 1**	0.697 (0.692-0.702)	0.692 (0.687-0.698)	0.668 (0.662-0.675)	0.723 (0.711-0.735)	0.715 (0.703-0.728)	0.686 (0.670-0.702)
2	*Model 2**	0.715 (0.708-0.720)	0.714 (0.709-0.720)	0.687 (0.680-0.693)	0.758 (0.744-0.771)	0.743 (0.731-0.755)	0.708 (0.692-0.724)
3	De Weerd et al, 2014[23] *Model 1*†	0.745 (0.739-0.751)	0.740 (0.735-0.745)	0.719 (0.713-0.724)	0.783 (0.770-0.795)	0.770 (0.759-0.781)	0.747 (0.733-0.761)
4	*Model 2*†	0.745 (0.739-0.751)	0.740 (0.735-0.745)	0.719 (0.713-0.724)	0.783 (0.770-0.795)	0.770 (0.759-0.781)	0.747 (0.733-0.761)
5	Jacobowitz et al, 2003[24]	0.673 (0.667-0.678)	0.668 (0.662-0.673)	0.644 (0.638-0.651)	0.689 (0.675-0.702)	0.680 (0.667-0.694)	0.647 (0.629-0.664)
6	Qureshi et al, 2001[25]	0.702 (0.696-0.707)	0.699 (0.694-0.704)	0.679 (0.673-0.685)	0.698 (0.686-0.710)	0.695 (0.683-0.707)	0.668 (0.652-0.683)

ACS indicates asymptomatic carotid artery stenosis; AUROC curve, area under receiver operating characteristic curve; CI, confidence interval; CVD, cardiovascular disease; TIA, transient ischemic attack.

* Model 1 refers to the model that was developed with predicted outcome 70-100% ACS and model 2 refers to the model that was developed with predicted outcome 50-100% ACS. † Model 1 refers to the model that was developed with predicted outcome >70% ACS and model 2 refers to the model that was developed with predicted outcome >50% ACS.

Figure S1. Calibration plots for outcome ≥70% ACS

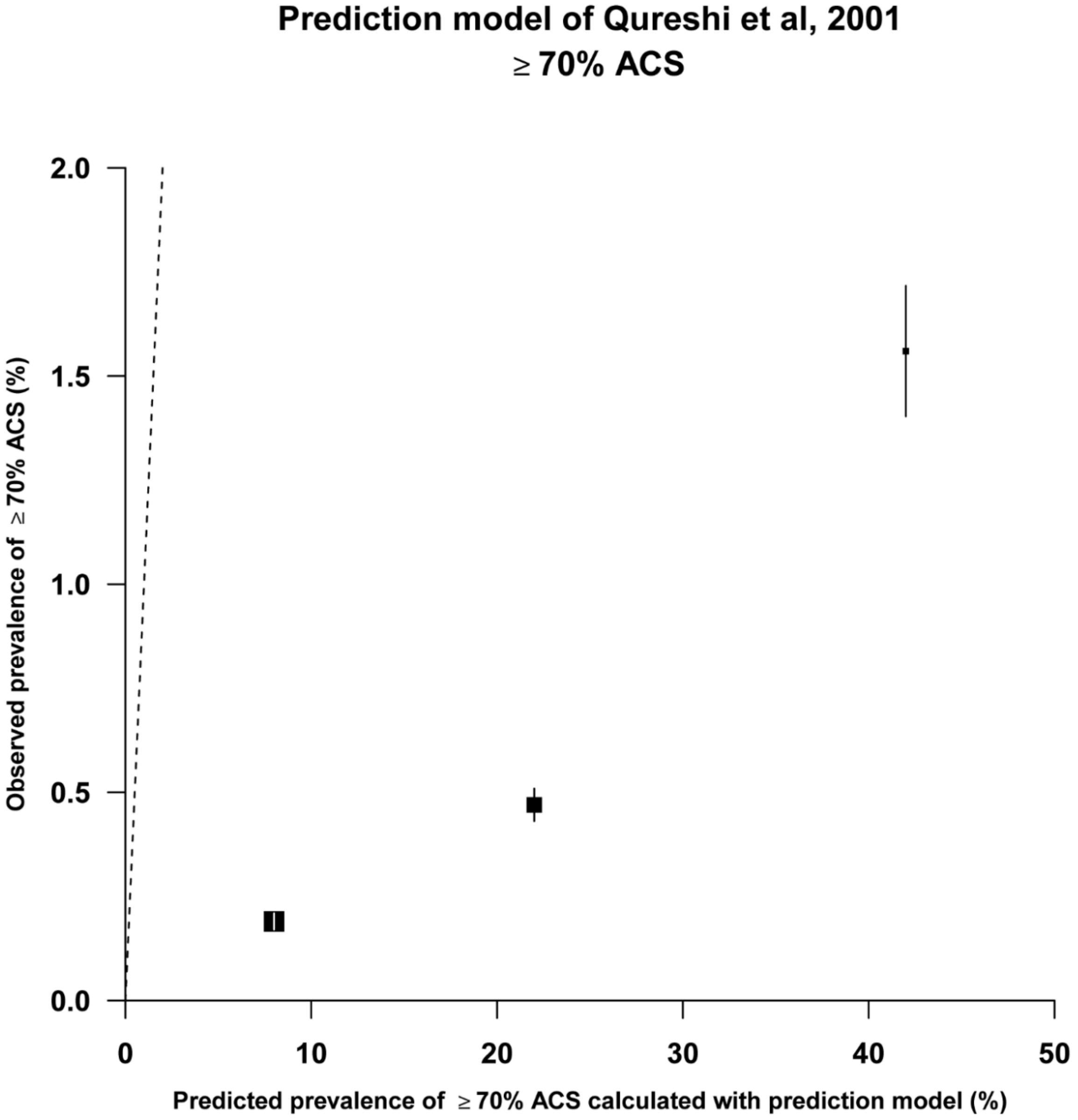

	Risk groups		
**Predicted prevalence (%)**	8	22	42
**Number of patients with ≥50% ACS**	702	960	371
**Observed prevalence in validation cohort (%)**	0.19	0.47	1.56

ACS indicates asymptomatic carotid artery stenosis.

Figure S2. A Calibration plot of external validation of the prediction model developed by Qureshi et al, 2001 (originally developed for ≥60% ACS).[25] It shows the predicted and observed prevalence of ≥70% ACS. The boxes represent the risk groups as provided in the original article and vertical lines represent the 95% confidence intervals.

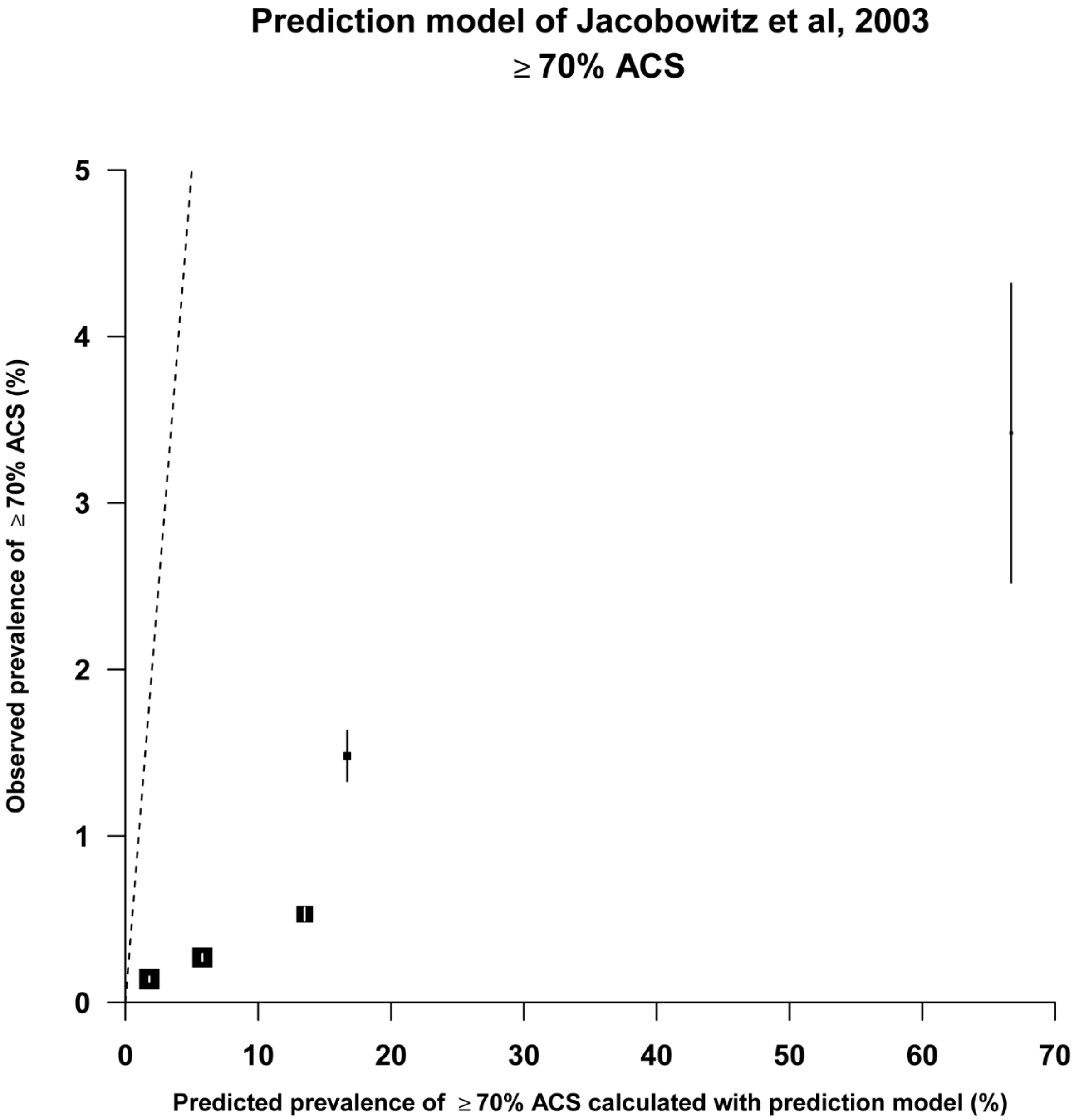

	Risk groups				
**Predicted prevalence (%)**	1.8	5.8	13.5	16.7	66.7
**Number of patients with ≥50% ACS**	308	585	724	362	54
**Observed prevalence in validation cohort (%)**	0.14	0.27	0.53	1.48	3.42

ACS indicates asymptomatic carotid artery stenosis.

Figure S2. B Calibration plot of external validation of the prediction model developed by Jacobowitz et al, 2003 (originally developed for >50% ACS).[24] It shows the predicted and observed prevalence of ≥70% ACS. The boxes represent the risk groups as provided in the original article and vertical lines represent the 95% confidence intervals.

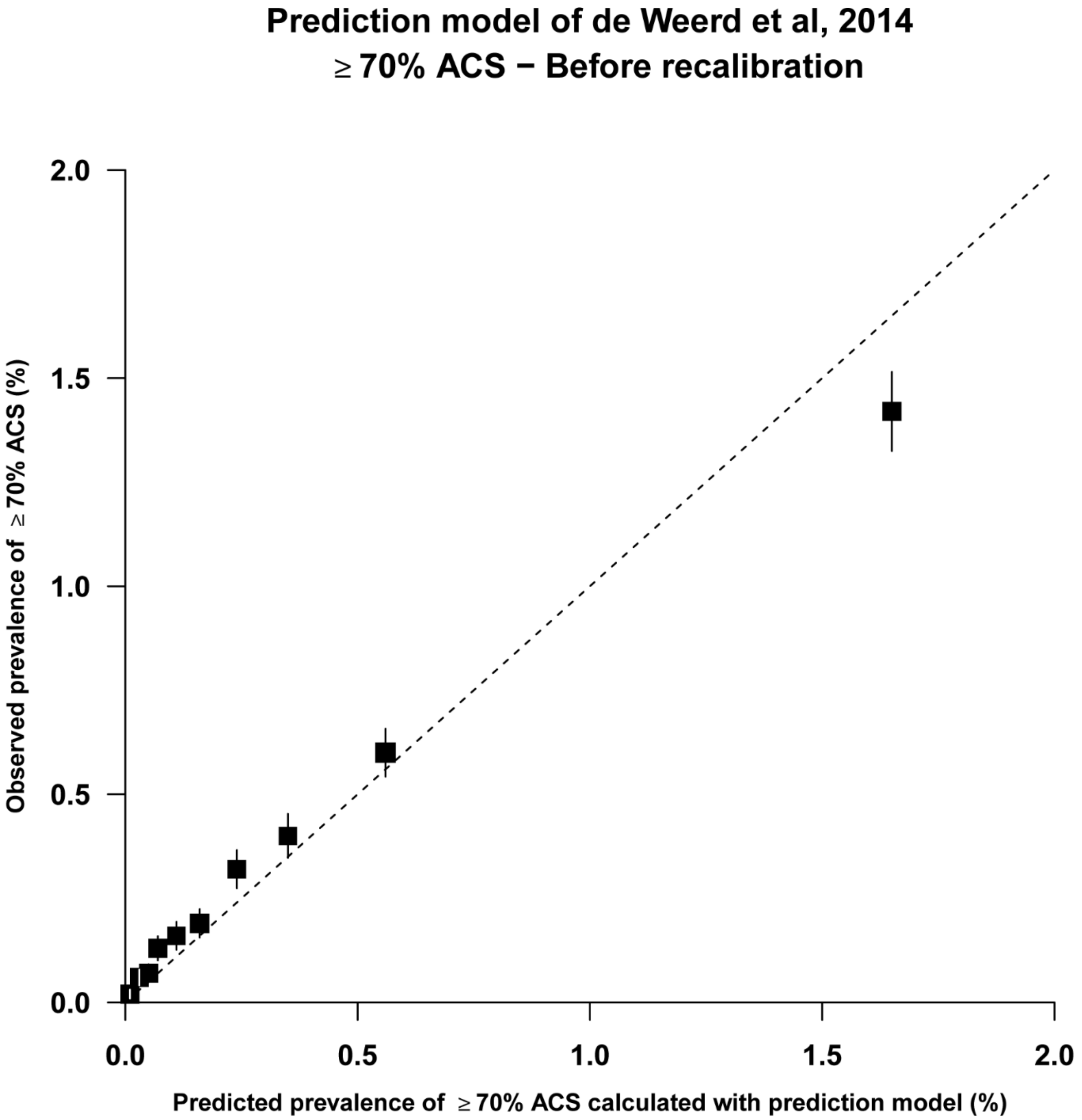

	Deciles of predicted risk									
**Predicted prevalence (%)**	0.01	0.03	0.05	0.07	0.11	0.16	0.24	0.35	0.56	1.65
**Number of patients with ≥50% ACS**	10	33	43	77	88	120	181	218	414	849
**Observed prevalence in validation cohort (%)**	0.02	0.06	0.07	0.13	0.16	0.19	0.32	0.40	0.60	1.42

ACS indicates asymptomatic carotid artery stenosis.

Figure S2. C Calibration plot of external validation of the prediction model (originally developed for ≥70% ACS) developed by de Weerd et al, 2014.[23] It shows the predicted and observed prevalence of ≥70% ACS (before recalibration). The boxes represent one decile of predicted risk and the vertical lines represent the 95% confidence intervals.

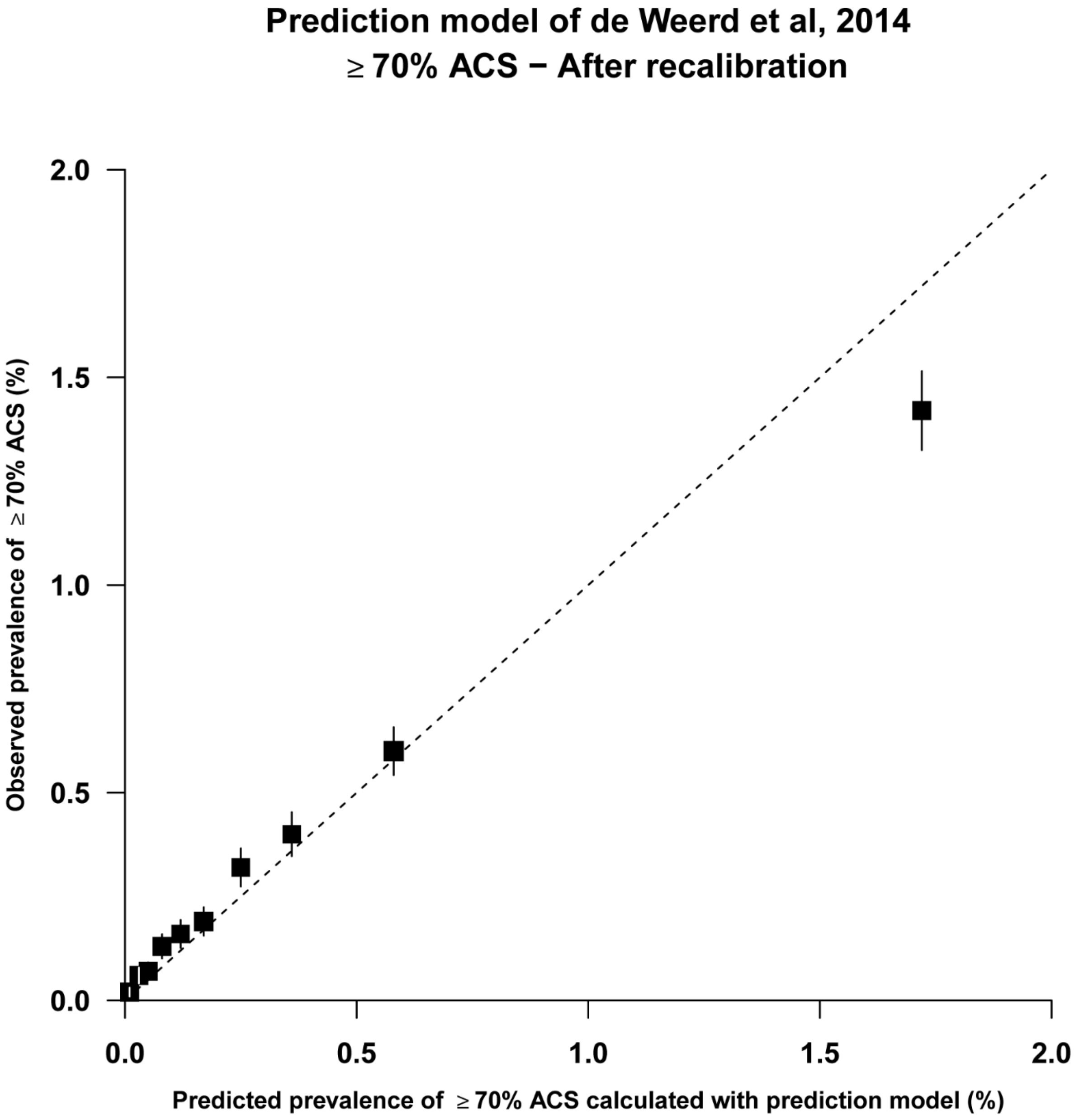

	Deciles of predicted risk									
**Predicted prevalence (%)**	0.01	0.03	0.05	0.08	0.12	0.17	0.25	0.36	0.58	1.72
**Number of patients with ≥50% ACS**	10	33	43	77	88	120	181	218	415	848
**Observed prevalence in validation cohort (%)**	0.02	0.06	0.07	0.13	0.16	0.19	0.32	0.40	0.60	1.42

ACS indicates asymptomatic carotid artery stenosis.

Figure S2. D Calibration plot of external validation of the prediction model for ≥70% ACS developed by de Weerd et al, 2014.[23] It shows the predicted and observed prevalence of ≥70% ACS (after recalibration with adjusting the intercept). The boxes represent one decile of predicted risk and the vertical lines represent the 95% confidence intervals.

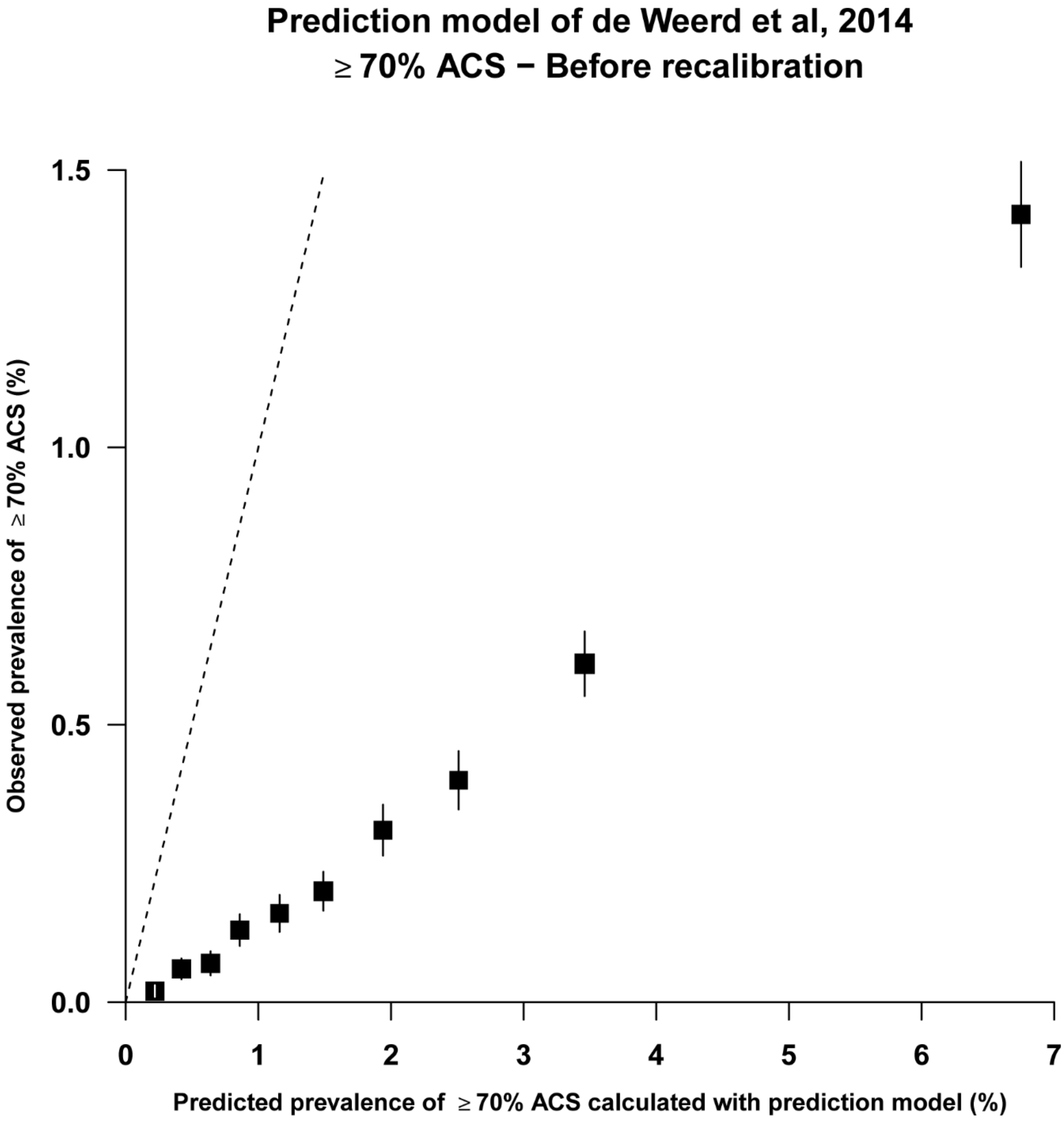

	Deciles of predicted risk									
**Predicted prevalence (%)**	0.22	0.42	0.64	0.86	1.16	1.49	1.94	2.51	3.46	6.75
**Number of patients with ≥50% ACS**	10	33	44	76	88	128	180	216	413	845
**Observed prevalence in validation cohort (%)**	0.02	0.06	0.07	0.13	0.16	0.20	0.31	0.40	0.61	1.42

ACS indicates asymptomatic carotid artery stenosis.

Figure S2. E Calibration plot of external validation of the prediction model (originally developed for ≥50% ACS) developed by de Weerd et al, 2014.[23] It shows the predicted and observed prevalence of ≥70% ACS (before recalibration). The boxes represent one decile of predicted risk and the vertical lines represent the 95% confidence intervals.

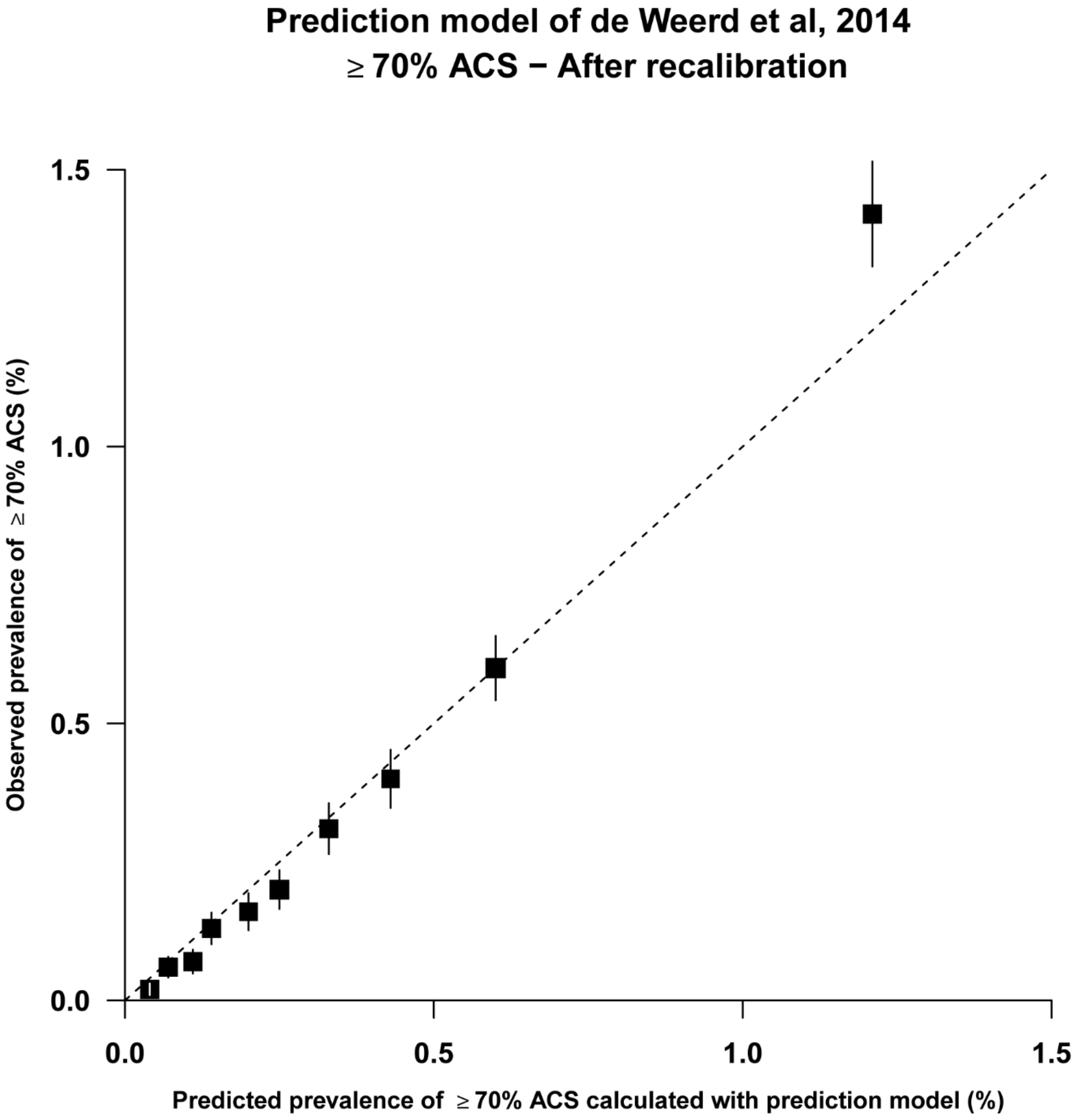

	Deciles of predicted risk									
**Predicted prevalence (%)**	0.04	0.07	0.11	0.14	0.20	0.25	0.33	0.43	0.60	1.21
**Number of patients with ≥50% ACS**	10	33	44	76	88	128	180	216	412	846
**Observed prevalence in validation cohort (%)**	0.02	0.06	0.07	0.13	0.16	0.20	0.31	0.40	0.60	1.42

ACS indicates asymptomatic carotid artery stenosis.

Figure S2. F Calibration plot of external validation of the prediction model (originally developed for ≥50% ACS) developed by de Weerd et al, 2014.[23] It shows the predicted and observed prevalence of ≥70% ACS (after recalibration with adjusting the intercept). The boxes represent one decile of predicted risk and the vertical lines represent the 95% confidence intervals.

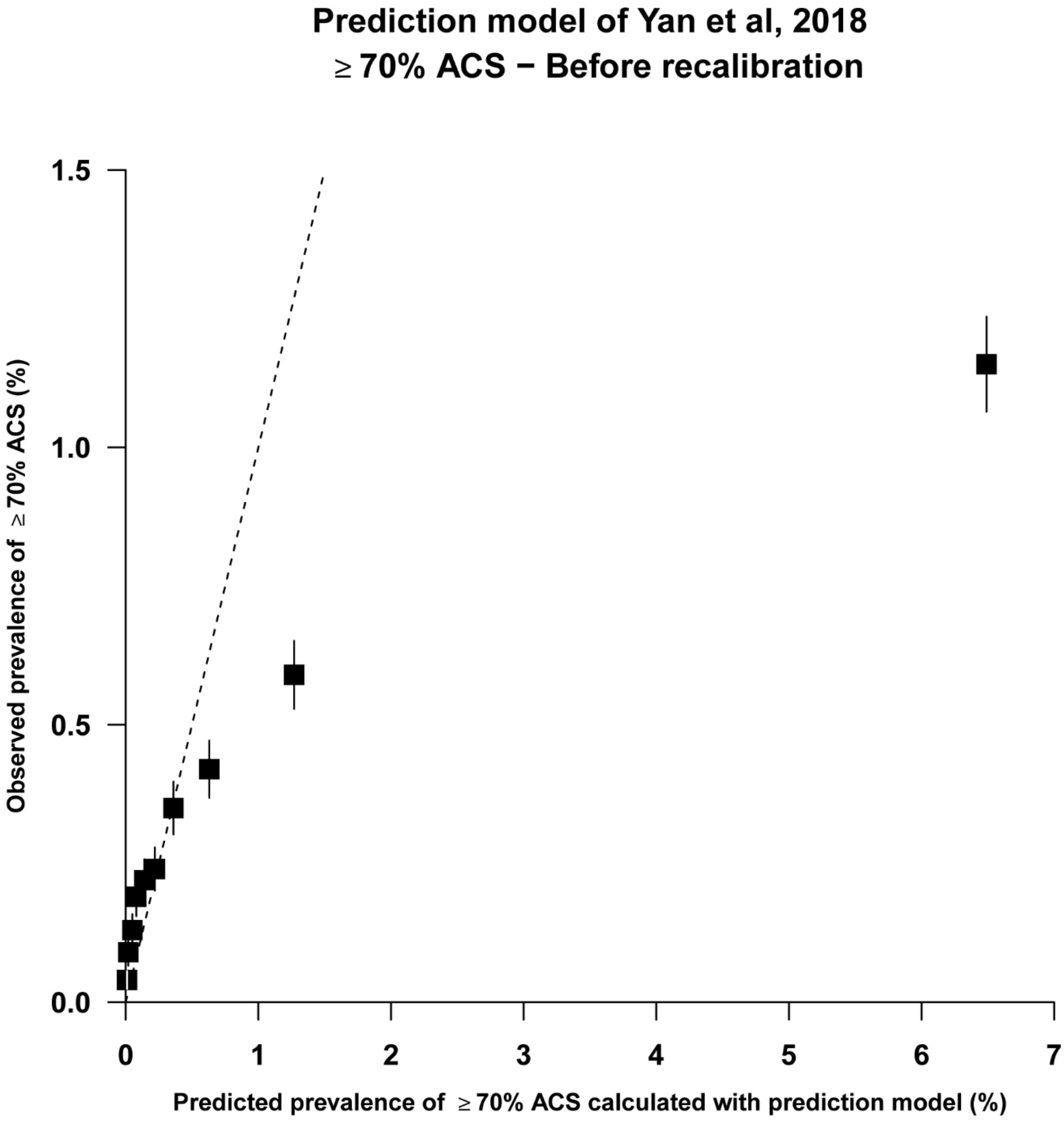

	Deciles of predicted risk									
**Predicted prevalence (%)**	0.01	0.02	0.05	0.08	0.14	0.22	0.36	0.63	1.27	6.49
**Number of patients with ≥50% ACS**	24	54	76	114	129	141	209	248	351	688
**Observed prevalence in validation cohort (%)**	0.04	0.09	0.13	0.19	0.22	0.24	0.35	0.42	0.59	1.15

ACS indicates asymptomatic carotid artery stenosis.

Figure S2. G. Calibration plot of external validation of the prediction model (originally developed for ≥70% ACS) developed by de Yan et al, 2018.[26] It shows the predicted and observed prevalence of ≥70% ACS (before recalibration). The boxes represent one decile of predicted risk and the vertical lines represent the 95% confidence intervals.

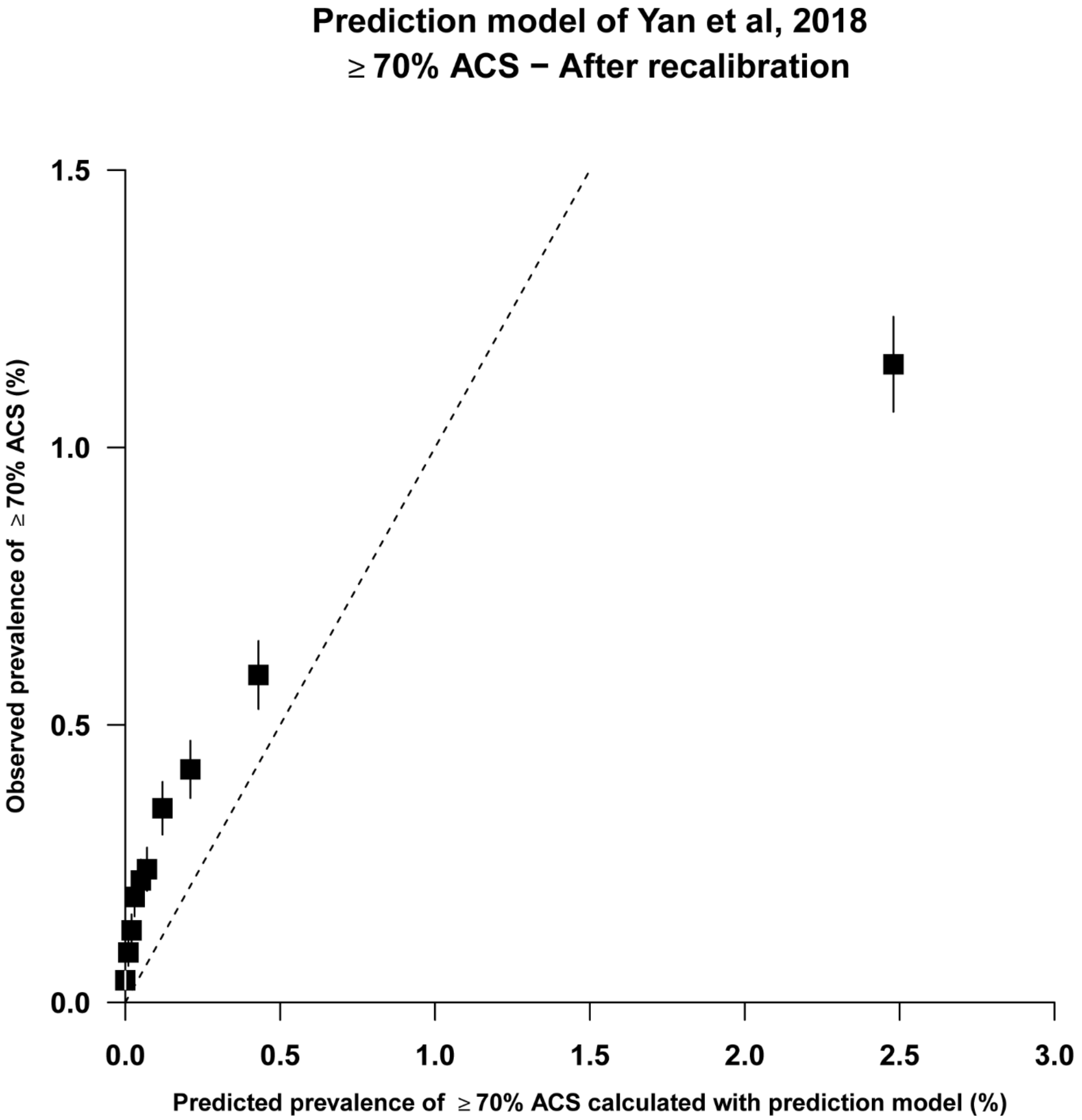

	Deciles of predicted risk									
**Predicted prevalence (%)**	0.00	0.01	0.02	0.03	0.05	0.07	0.12	0.21	0.43	2.48
**Number of patients with ≥50% ACS**	24	54	76	114	129	141	209	248	351	687
**Observed prevalence in validation cohort (%)**	0.04	0.09	0.13	0.19	0.22	0.24	0.35	0.42	0.59	1.15

ACS indicates asymptomatic carotid artery stenosis.

Figure S2. H Calibration plot of external validation of the prediction model (originally developed for ≥70% ACS) developed by de Yan et al, 2018.[26] It shows the predicted and observed prevalence of ≥70% ACS (after recalibration with adjusting the intercept). The boxes represent one decile of predicted risk and the vertical lines represent the 95% confidence intervals.

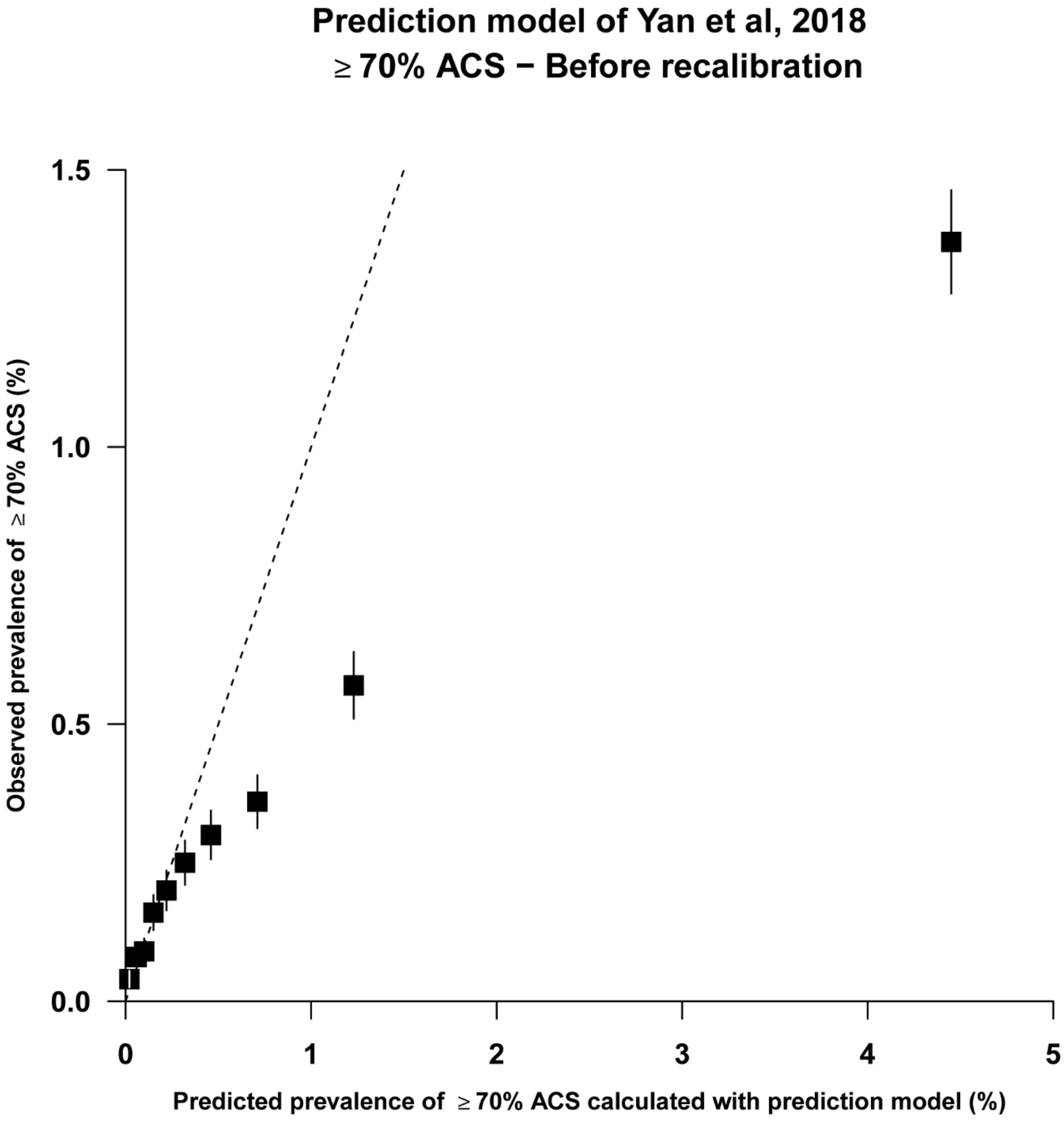

	Deciles of predicted risk									
**Predicted prevalence (%)**	0.02	0.06	0.10	0.15	0.22	0.32	0.46	0.71	1.23	4.45
**Number of patients with ≥50% ACS**	21	49	51	93	120	149	180	214	338	818
**Observed prevalence in validation cohort (%)**	0.04	0.08	0.09	0.16	0.20	0.25	0.30	0.36	0.57	1.37

ACS indicates asymptomatic carotid artery stenosis.

Figure S2. I Calibration plot of external validation of the prediction model (originally developed for ≥50% ACS) developed by de Yan et al, 2018.[26] It shows the predicted and observed prevalence of ≥70% ACS (before recalibration). The boxes represent one decile of predicted risk and the vertical lines represent the 95% confidence intervals.

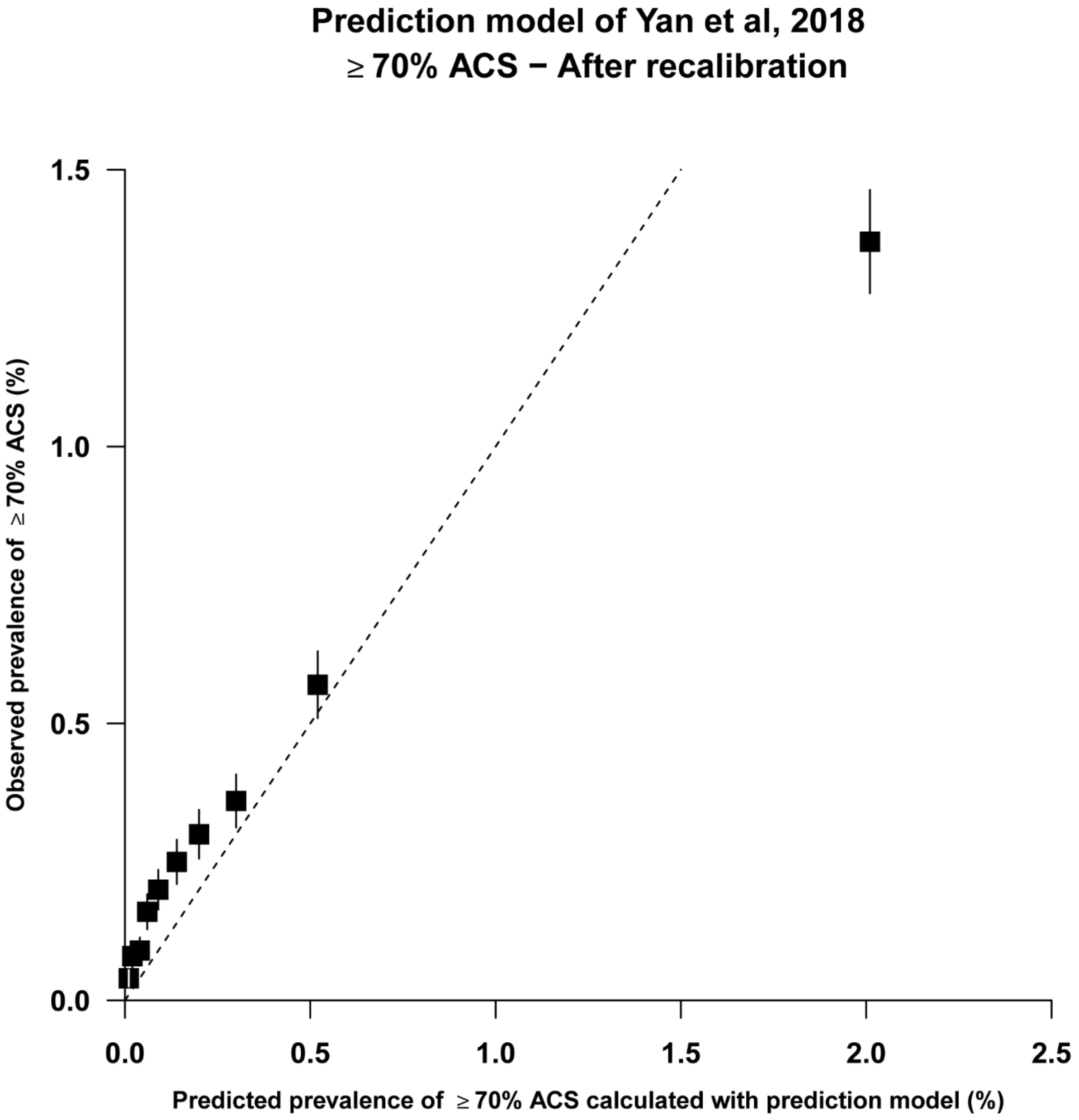

	Deciles of predicted risk									
**Predicted prevalence (%)**	0.01	0.02	0.04	0.06	0.09	0.14	0.20	0.30	0.52	2.01
**Number of patients with ≥50% ACS**	21	49	51	93	120	149	180	214	338	818
**Observed prevalence in validation cohort (%)**	0.04	0.08	0.09	0.16	0.20	0.25	0.30	0.35	0.57	1.37

ACS indicates asymptomatic carotid artery stenosis.

Figure S2. J Calibration plot of external validation of the prediction model (originally developed for ≥50% ACS) developed by de Yan et al, 2018.[26] It shows the predicted and observed prevalence of ≥70% ACS (after recalibration with adjusting the intercept). The boxes represent one decile of predicted risk and the vertical lines represent the 95% confidence intervals.

Figure S2. Calibration plots for outcome ≥50% ACS

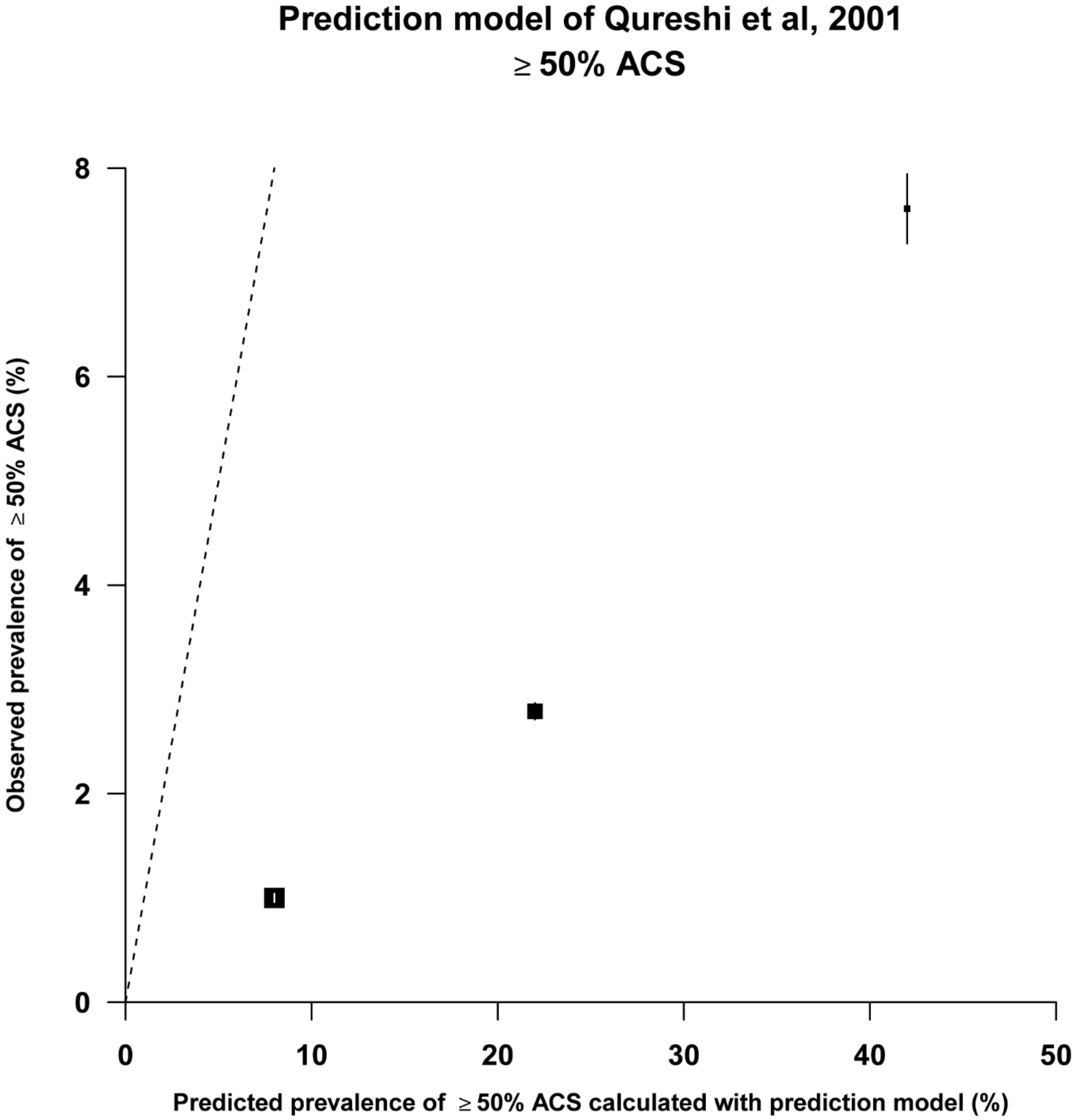

	Risk groups		
**Predicted prevalence (%)**	8	22	42
**Number of patients with ≥50% ACS**	3713	5658	1807
**Observed prevalence in validation cohort (%)**	1.00	2.79	7.61

ACS indicates asymptomatic carotid artery stenosis.

Figure S2. A Calibration plot of external validation of the prediction model developed by Qureshi et al, 2001 (originally developed for ≥60% ACS).[25] It shows the predicted and observed prevalence of ≥50% ACS. The boxes represent the risk groups as provided in the original article and vertical lines represent the 95% confidence intervals.

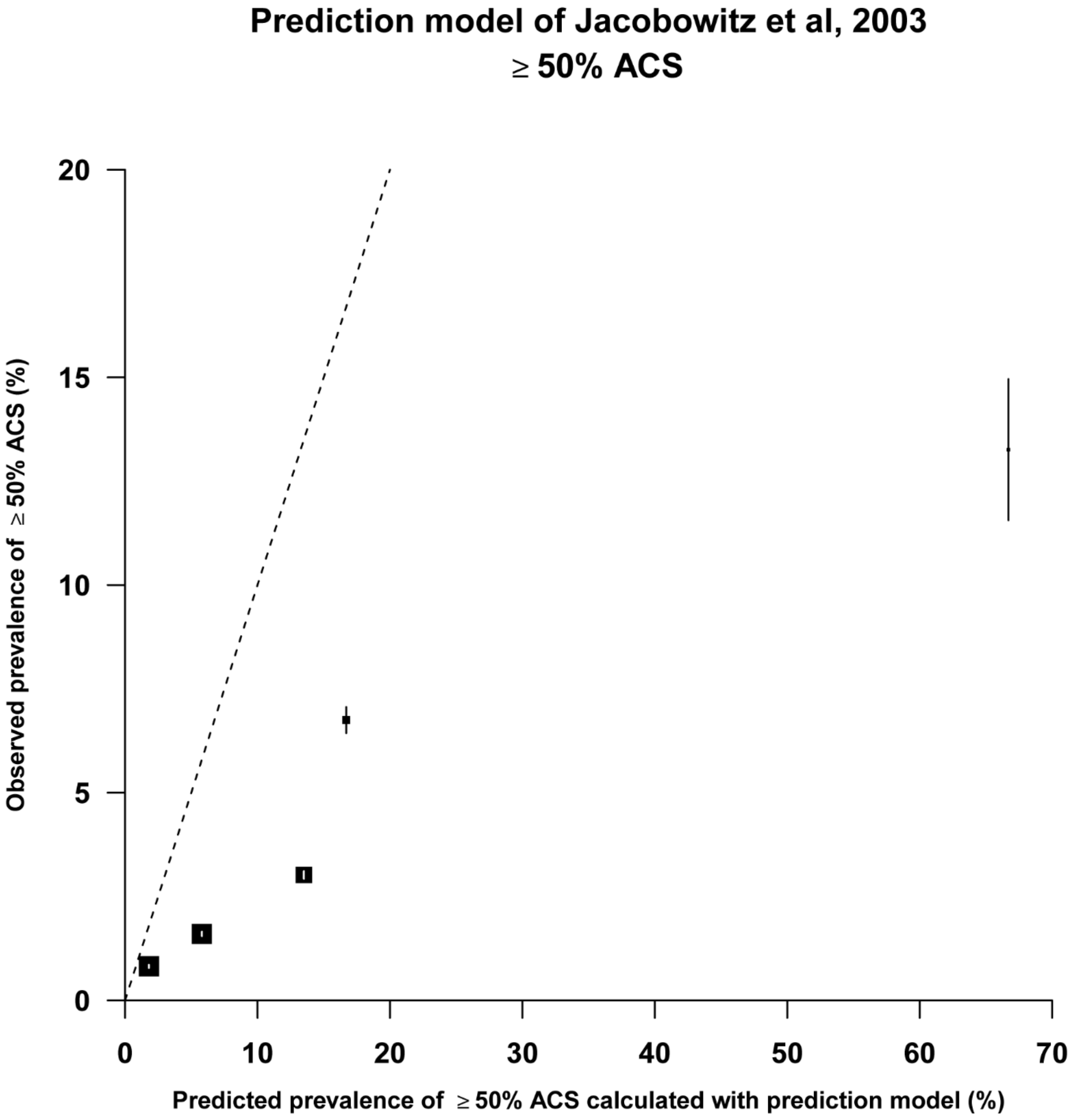

	Risk groups				
**Predicted prevalence (%)**	1.8	5.8	13.5	16.7	66.7
**Number of patients with ≥50% ACS**	1798	3419	4100	1658	203
**Observed prevalence in validation cohort (%)**	0.82	1.60	3.02	6.75	13.26

ACS indicates asymptomatic carotid artery stenosis.

Figure S2. B Calibration plot of external validation of the prediction model developed by Jacobowitz et al, 2003 (originally developed for >50% ACS).[24] It shows the predicted and observed prevalence of ≥50% ACS. The boxes represent the risk groups as provided in the original article and vertical lines represent the 95% confidence intervals.

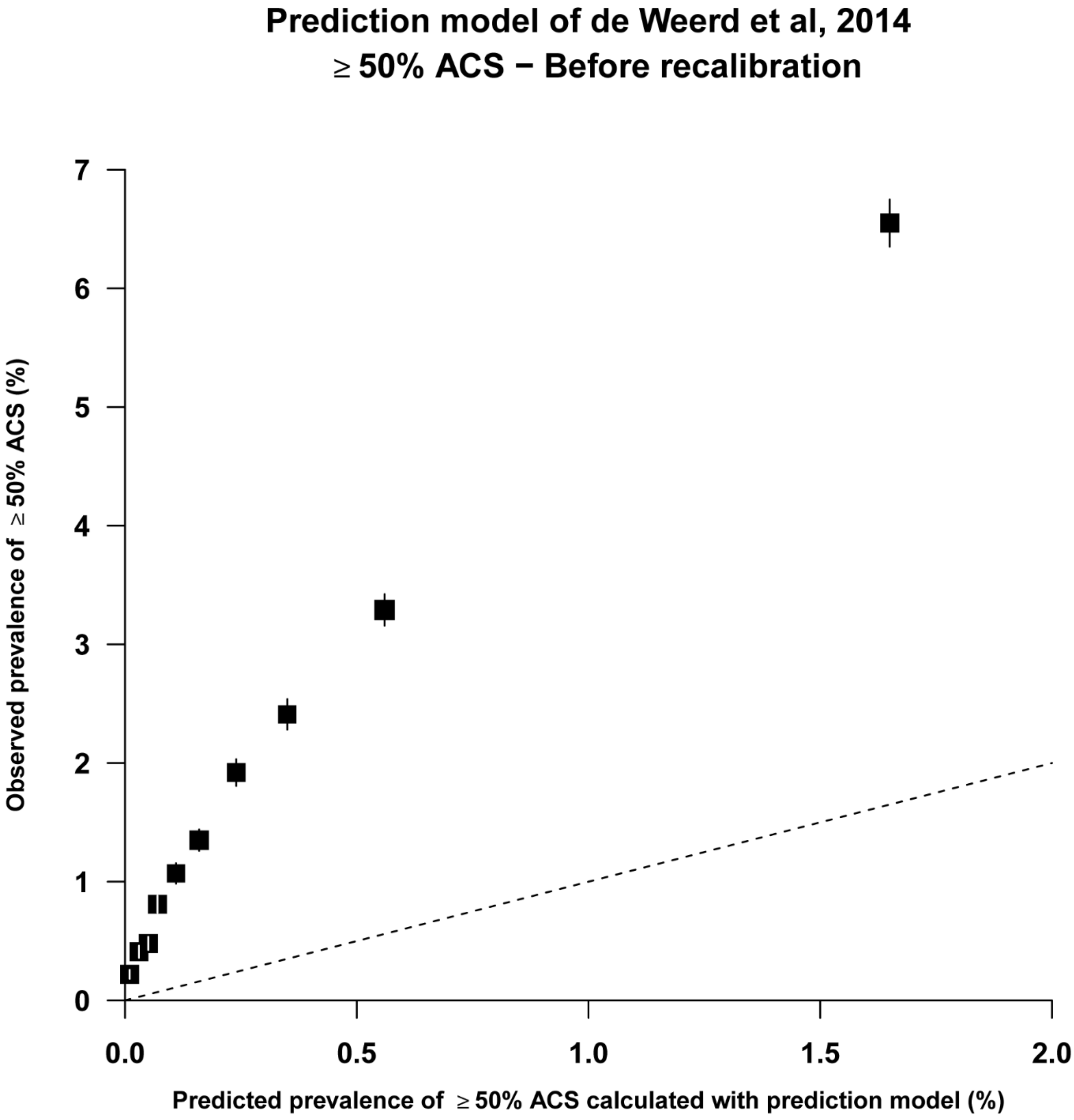

	Deciles of predicted risk									
**Predicted prevalence (%)**	0.01	0.03	0.05	0.07	0.11	0.16	0.24	0.35	0.56	1.65
**Number of patients with ≥50% ACS**	130	246	285	480	583	851	1103	1316	2273	3911
**Observed prevalence in validation cohort (%)**	0.22	0.41	0.48	0.81	1.07	1.35	1.92	2.41	3.29	6.55

ACS indicates asymptomatic carotid artery stenosis.

Figure S2. C Calibration plot of external validation of the prediction model developed by de Weerd et al, 2014 (originally developed for ≥70% ACS).[23] It shows the predicted and observed prevalence of ≥50% ACS (before recalibration). The boxes represent one decile of predicted risk and the vertical lines represent the 95% confidence intervals.

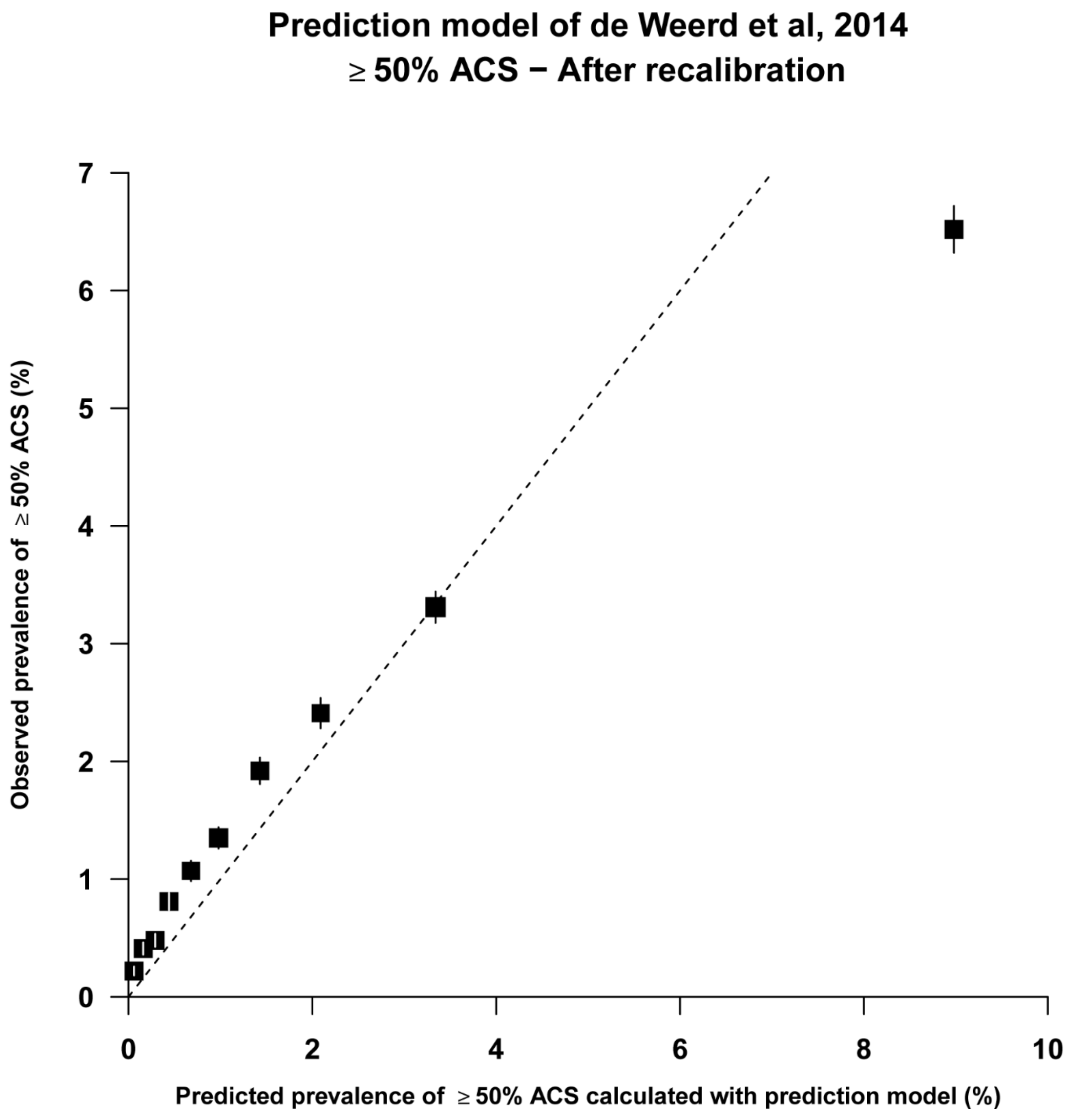

	Deciles of predicted risk									
**Predicted prevalence (%)**	0.06	0.16	0.29	0.44	0.68	0.98	1.43	2.09	3.34	8.98
**Number of patients with ≥50% ACS**	130	246	285	480	584	851	1103	1317	2291	3891
**Observed prevalence in validation cohort (%)**	0.22	0.41	0.48	0.81	1.07	1.35	1.92	2.41	3.31	6.52

ACS indicates asymptomatic carotid artery stenosis.

Figure S2. D Calibration plot of external validation of the prediction model developed by de Weerd et al, 2014 (originally developed for ≥70% ACS).[23] It shows the predicted and observed prevalence of ≥50% ACS (after recalibration with adjusting the intercept). The boxes represent one decile of predicted risk and the vertical lines represent the 95% confidence intervals.

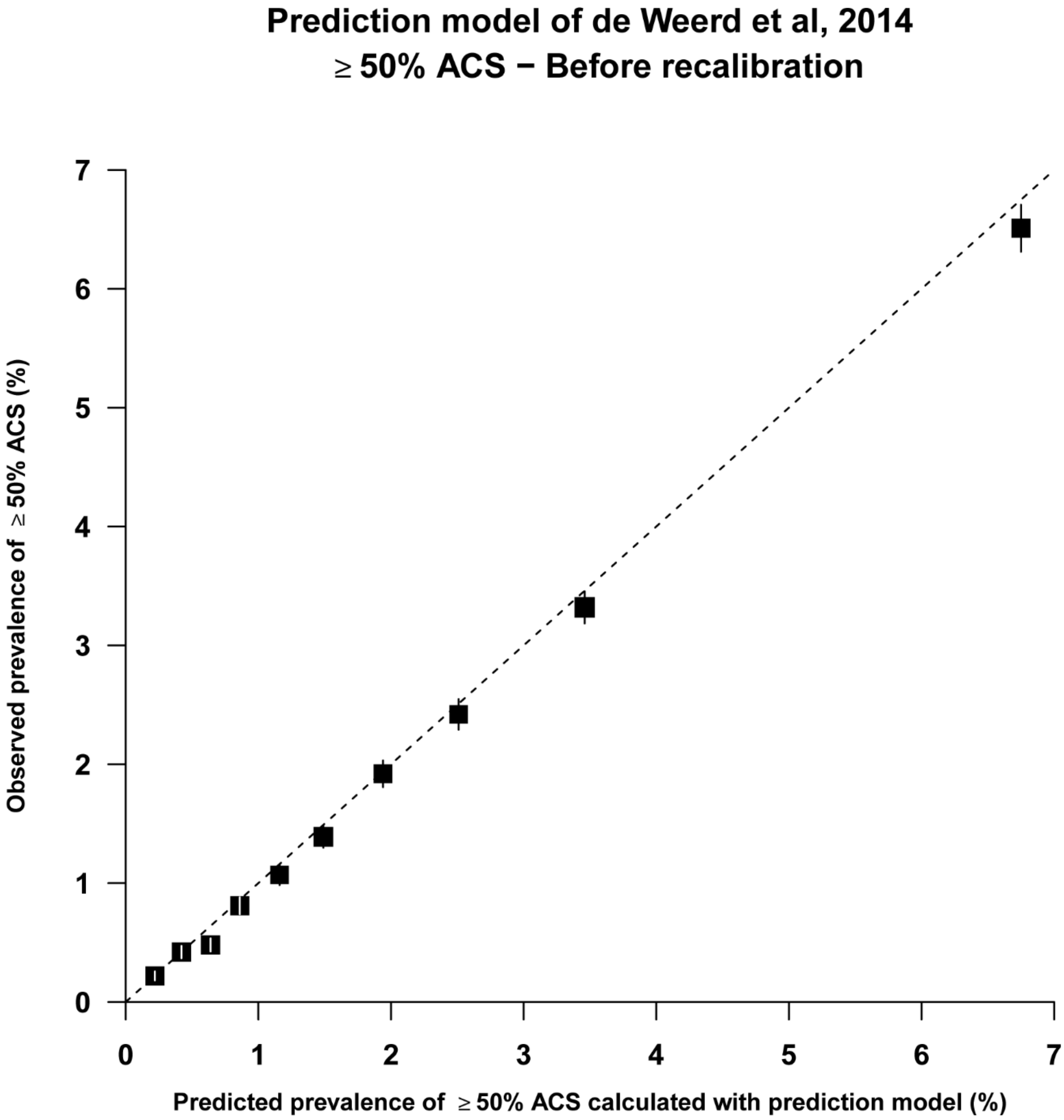

	Deciles of predicted risk									
**Predicted prevalence (%)**	0.22	0.42	0.64	0.86	1.16	1.49	1.94	2.51	3.46	6.75
**Number of patients with ≥50% ACS**	130	251	284	480	589	879	1096	1320	2263	3886
**Observed prevalence in validation cohort (%)**	0.22	0.42	0.48	0.81	1.07	1.39	1.92	2.42	3.32	6.51

ACS indicates asymptomatic carotid artery stenosis.

Figure S2. E Calibration plot of external validation of the prediction model for ≥50% ACS developed by de Weerd et al, 2014.[23] It shows the predicted and observed prevalence of ≥50% ACS (before recalibration). The boxes represent one decile of predicted risk and the vertical lines represent the 95% confidence intervals.

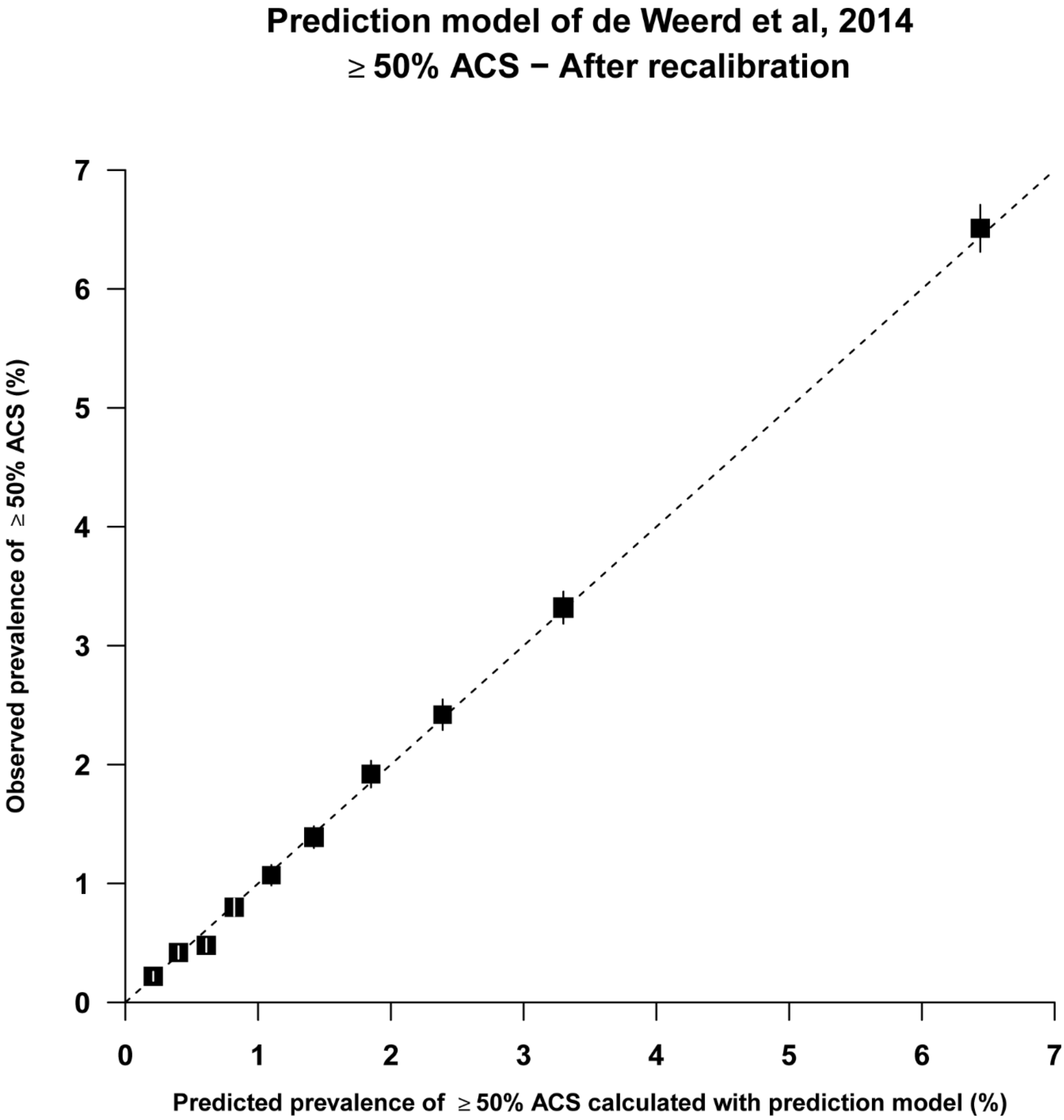

	Deciles of predicted risk									
**Predicted prevalence (%)**	0.21	0.40	0.61	0.82	1.10	1.42	1.85	2.39	3.30	6.44
**Number of patients with ≥50% ACS**	131	250	284	480	589	879	1096	1320	2264	3885
**Observed prevalence in validation cohort (%)**	0.22	0.42	0.48	0.80	1.07	1.39	1.92	2.42	3.32	6.51

ACS indicates asymptomatic carotid artery stenosis.

Figure S2 F Calibration plot of external validation of the prediction model for ≥50% ACS developed by de Weerd et al, 2014.[23] It shows the predicted and observed prevalence of ≥50% ACS (after recalibration). The boxes represent one decile of predicted risk and the vertical lines represent the 95% confidence intervals. Figure also shown as Figure 3A in the manuscript.

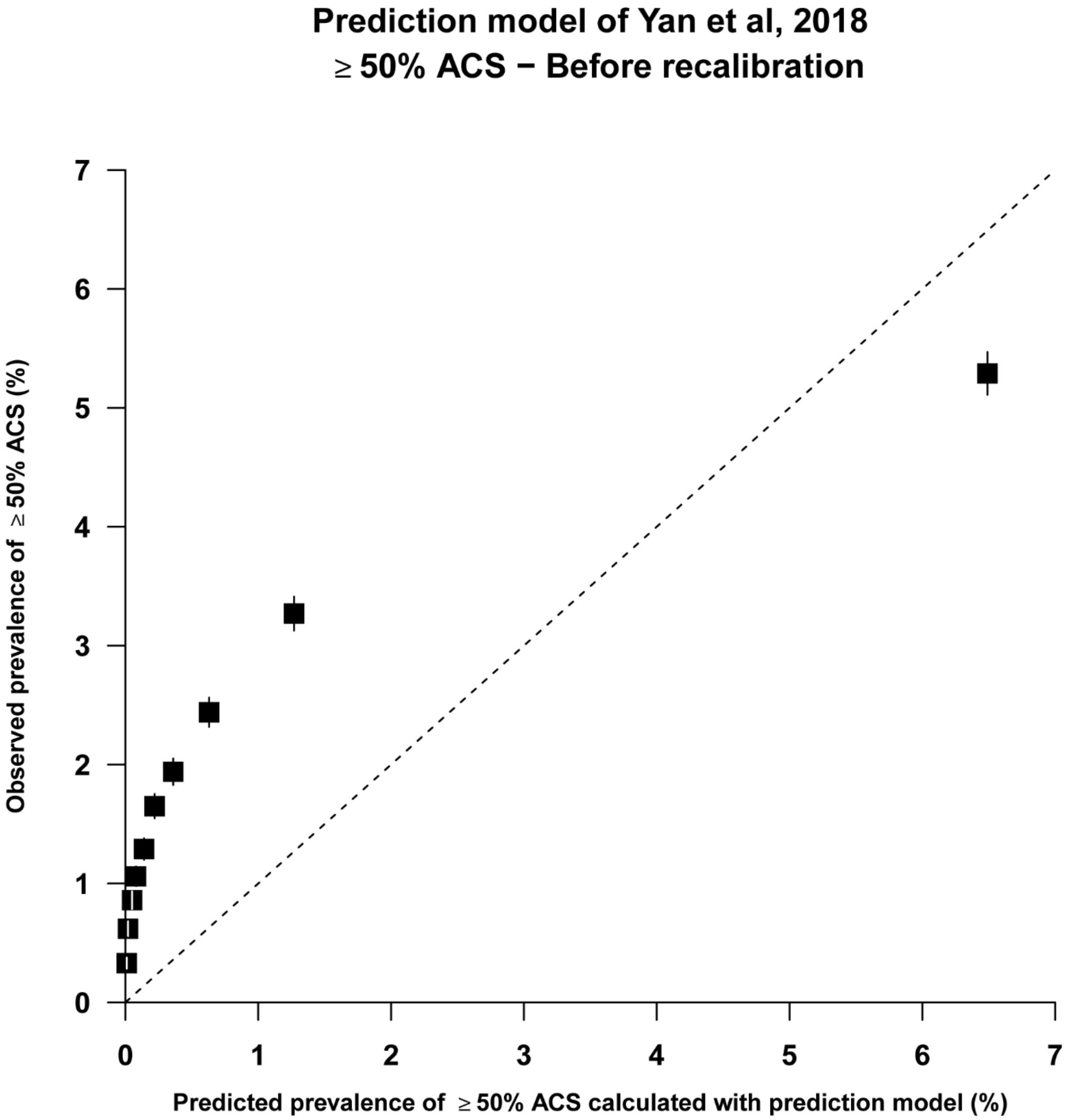

	Deciles of predicted risk									
**Predicted prevalence (%)**	0.01	0.02	0.05	0.08	0.14	0.22	0.36	0.63	1.27	6.49
**Number of patients with ≥50% ACS**	198	368	511	633	771	983	1156	1453	1952	3153
**Observed prevalence in validation cohort (%)**	0.33	0.62	0.86	1.06	1.29	1.65	1.94	2.44	3.27	5.29

ACS indicates asymptomatic carotid artery stenosis.

Figure S2 G Calibration plot of external validation of the prediction model developed by Yan et al, 2018 (originally developed for ≥70% ACS).[26] It shows the predicted and observed prevalence of ≥50% ACS (before recalibration). The boxes represent one decile of predicted risk and the vertical lines represent the 95% confidence intervals.

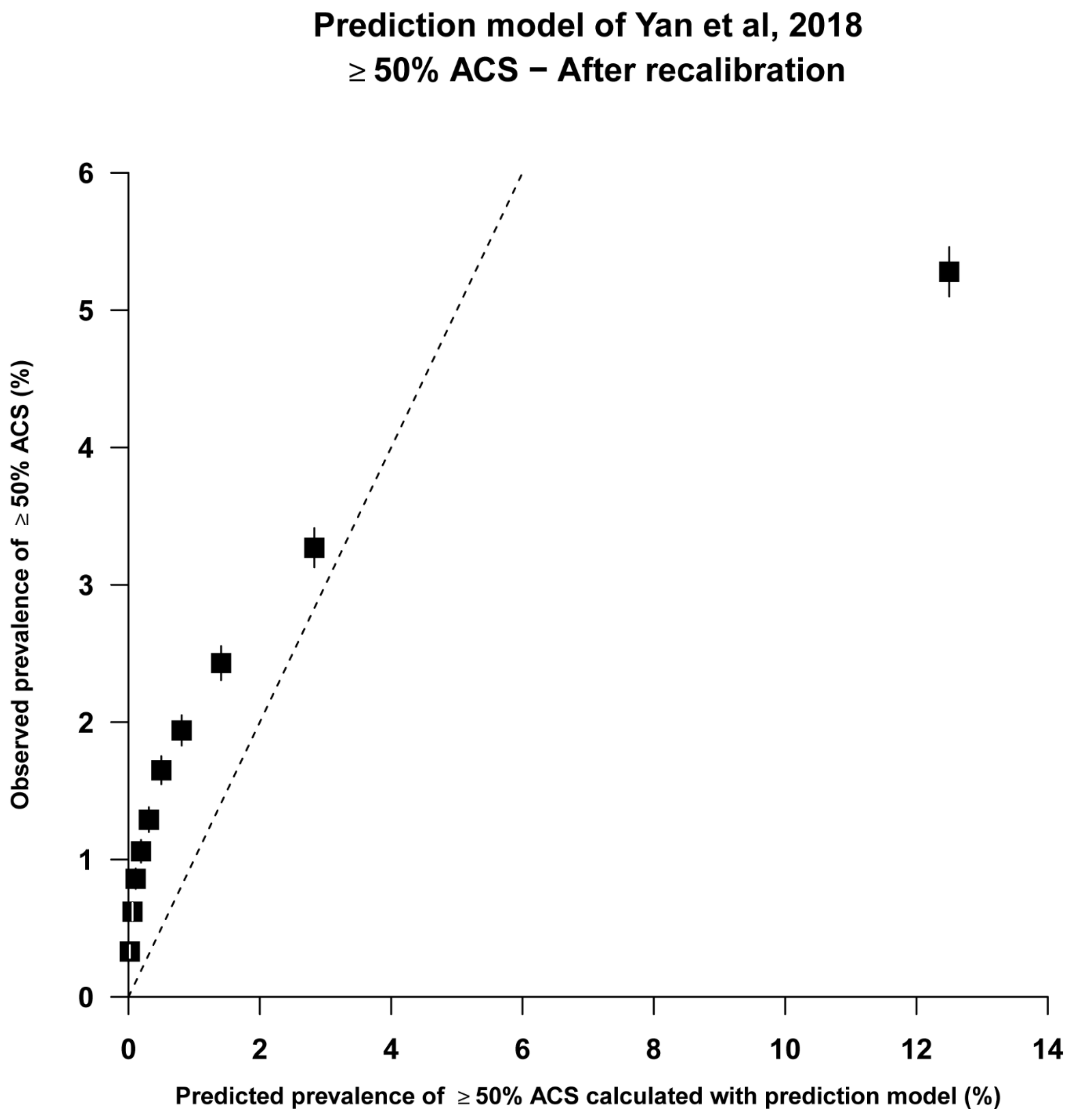

	Deciles of predicted risk									
**Predicted prevalence (%)**	0.02	0.06	0.11	0.19	0.31	0.50	0.81	1.41	2.83	12.50
**Number of patients with ≥50% ACS**	198	369	511	632	772	983	1156	1452	1953	3152
**Observed prevalence in validation cohort (%)**	0.33	0.62	0.86	1.06	1.29	1.65	1.94	2.43	3.27	5.28

ACS indicates asymptomatic carotid artery stenosis.

Figure S2 H Calibration plot of external validation of the prediction model developed by de Yan et al, 2018 (originally developed for ≥70% ACS).[26] It shows the predicted and observed prevalence of ≥50% ACS (after recalibration with adjusting the intercept). The boxes represent one decile of predicted risk and the vertical lines represent the 95% confidence intervals.

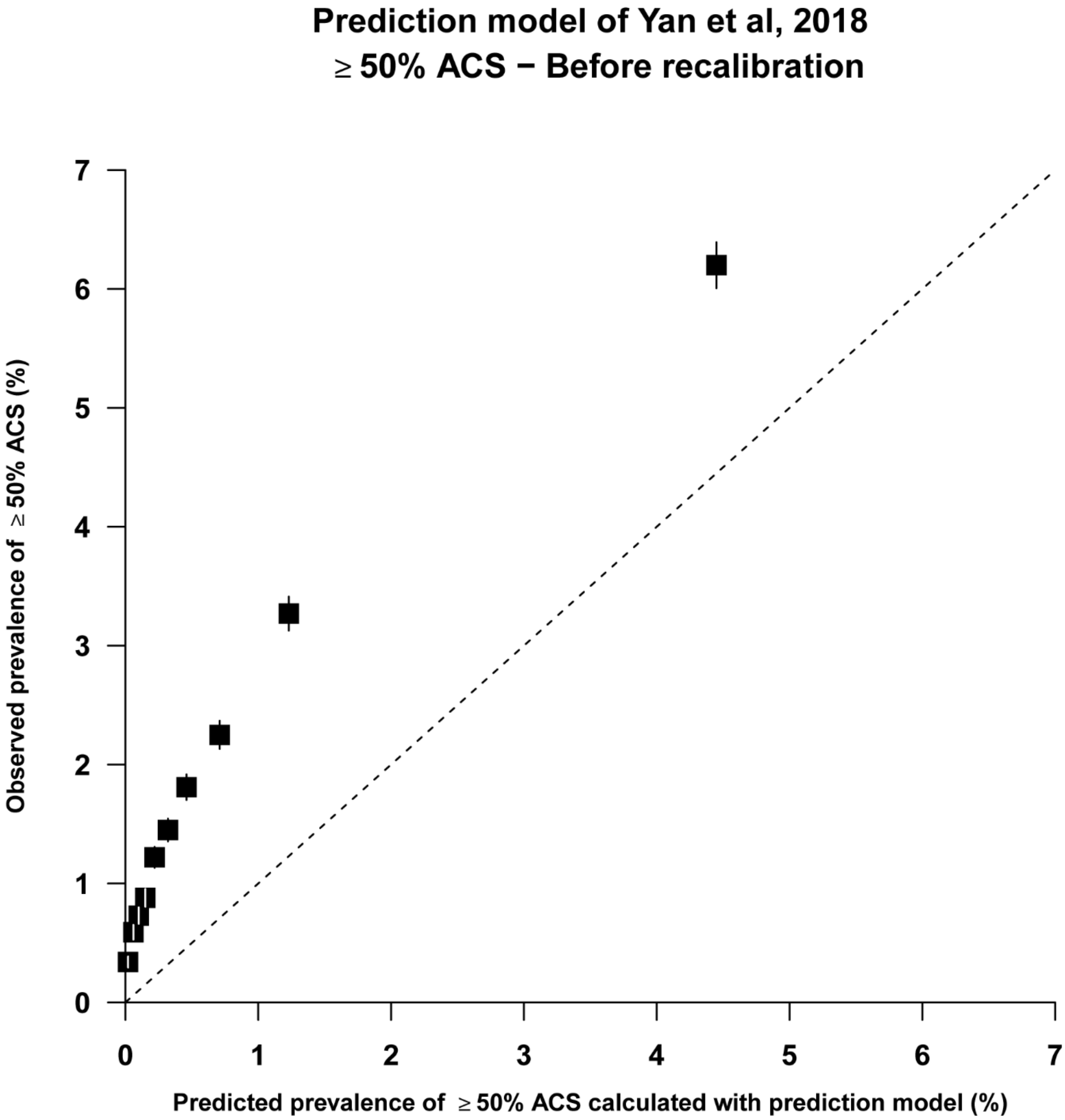

	Deciles of predicted risk									
**Predicted prevalence (%)**	0.02	0.06	0.10	0.15	0.22	0.32	0.46	0.71	1.23	4.45
**Number of patients with ≥50% ACS**	202	351	438	523	729	863	1081	1345	1950	3696
**Observed prevalence in validation cohort (%)**	0.34	0.59	0.73	0.88	1.22	1.45	1.81	2.25	3.27	6.20

ACS indicates asymptomatic carotid artery stenosis.

Figure S2 I Calibration plot of external validation of the prediction model for ≥50% developed by de Yan et al, 2018.[26] It shows the predicted and observed prevalence of ≥50% ACS (before recalibration). The boxes represent one decile of predicted risk and the vertical lines represent the 95% confidence intervals.

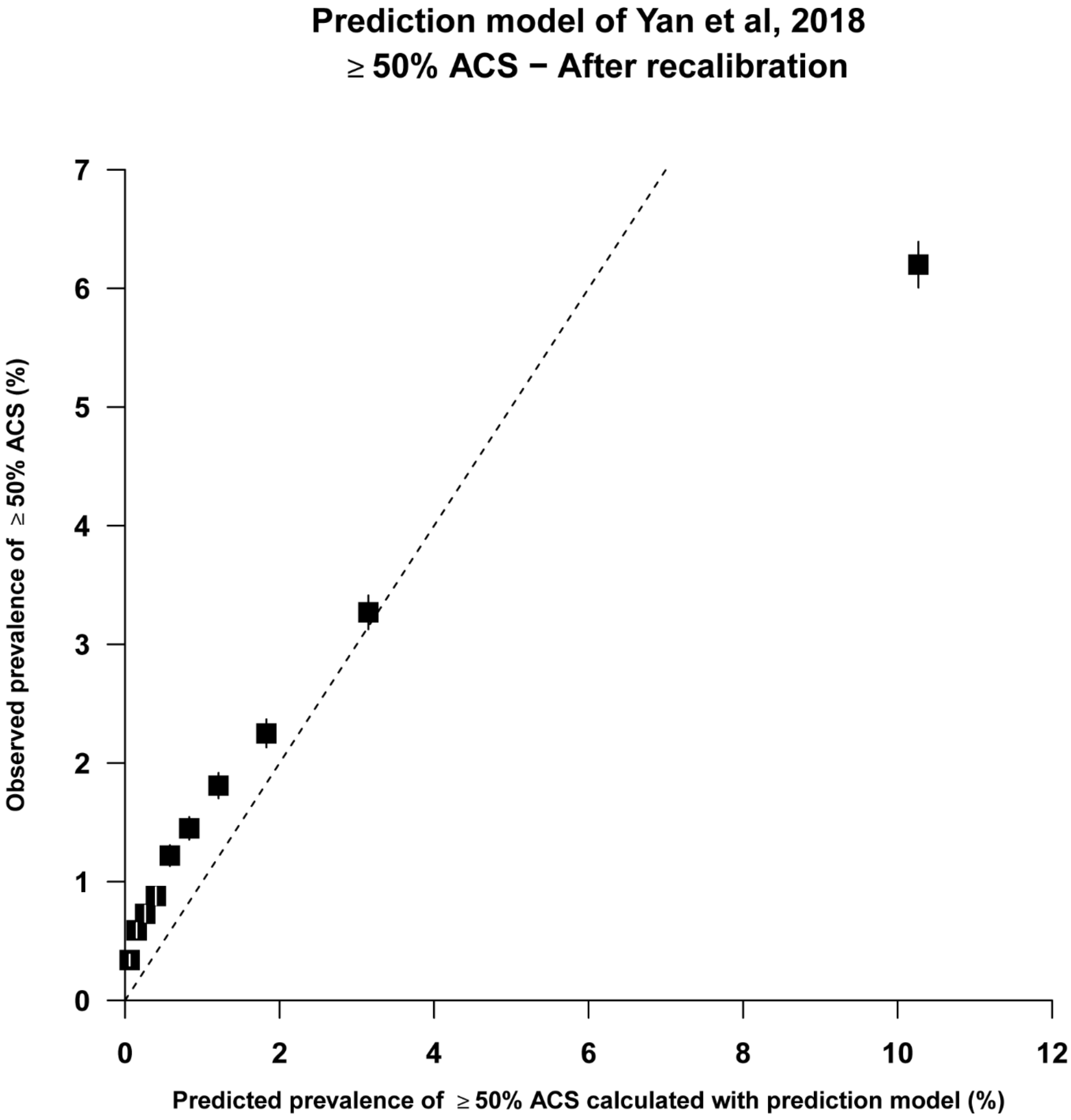

	Deciles of predicted risk									
**Predicted prevalence (%)**	0.06	0.15	0.26	0.40	0.58	0.83	1.21	1.83	3.15	10.27
**Number of patients with ≥50% ACS**	202	351	437	524	729	863	1082	1344	1948	3698
**Observed prevalence in validation cohort (%)**	0.34	0.59	0.73	0.88	1.22	1.45	1.81	2.25	3.27	6.20

ACS indicates asymptomatic carotid artery stenosis.

Figure S2 J Calibration plot of external validation of the prediction model for ≥50% ACS developed by Yan et al, 2018.[26] It shows the predicted and observed prevalence of ≥50% ACS (after recalibration with adjusting the intercept). The boxes represent one decile of predicted risk and the vertical lines represent the 95% confidence intervals.

Figure S3. Clinical application of the prediction model with the best discrimination

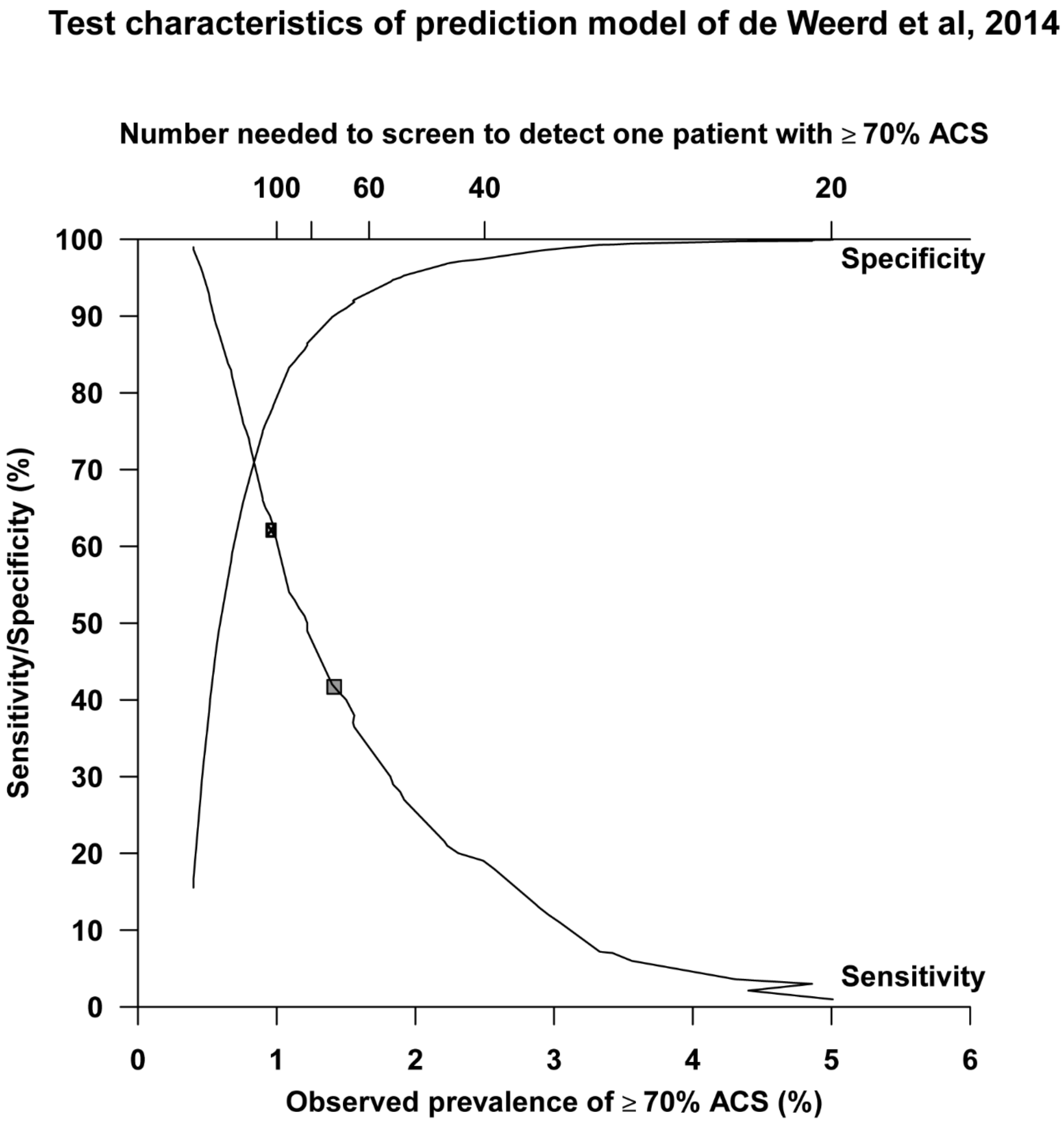

Graph showing the sensitivity and specificity and corresponding observed prevalence and number needed to screen to detect one patient with ≥70% ACS using the prediction model developed by de Weerd et al, 2014.[23] The square corresponds to targeted screening of patients in the highest decile of predicted risk. The prevalence in this decile is 1.42% with a number needed to screen of 70 and sensitivity is 41.7%. The circle corresponds to targeted screening of patients in the highest two deciles of predicted risk. The prevalence in these deciles is 0.98% with a number needed to screen of 102 and sensitivity of 62.1%.

Figure S4. Sensitivity analyses

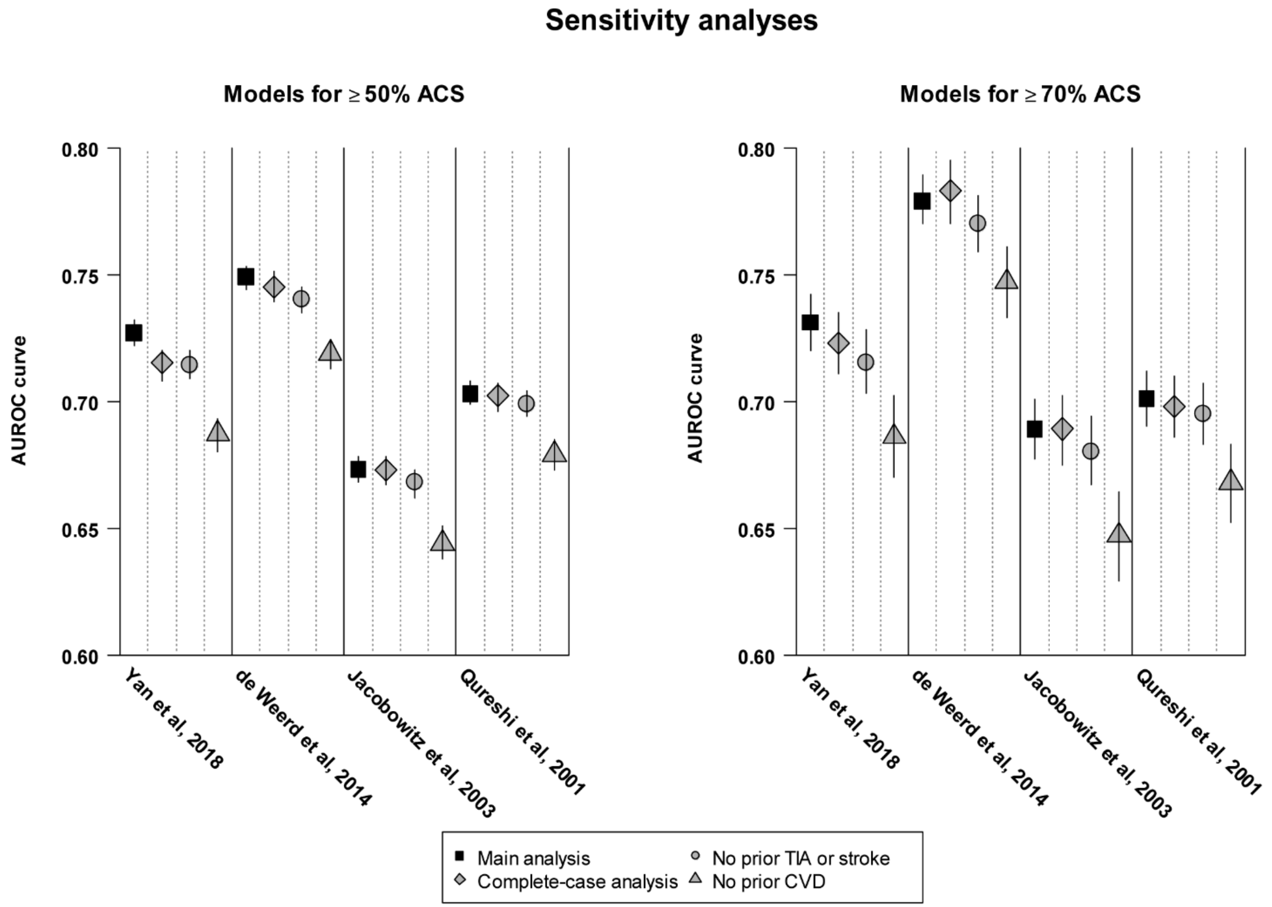

The boxes represent the AUROC curve of the analyses and the vertical lines represent the 95% confidence intervals.

ACS indicates asymptomatic carotid artery stenosis; AUROC curve, area under receiver operating characteristic curve.
